# Identification and characterization of L- and D-lactate-inducible systems from *Escherichia coli* MG1655, *Cupriavidus necator* H16 and *Pseudomonas* species

**DOI:** 10.1038/s41598-022-06028-7

**Published:** 2022-02-08

**Authors:** Ernesta Augustiniene, Naglis Malys

**Affiliations:** 1 Bioprocess Research Centre, Faculty of Chemical Technology, Kaunas University of Technology, Radvilėnų pl. 19, Kaunas, LT-50254 Lithuania; 2 Department of Organic Chemistry, Faculty of Chemical Technology, Kaunas University of Technology, Radvilėnų pl. 19, Kaunas, LT-50254 Lithuania

**Keywords:** Biotechnology, Genetics, Molecular biology

## Abstract

Lactic acid is an important platform chemical used for the production of various compounds including polylactic acid (PLA). Optically pure L- and D-lactic acids are required to obtain high quality PLA. To advance the development and selection of microbial strains for improved production of lactic acid enantiomers, a high-throughput screening, dynamic pathway control, or real-time monitoring are often applied. Inducible gene expression systems and their application in the genetically encoded biosensors contribute to the development of these techniques and are important devices for the advancement of lactic acid biotechnology. Here, we identify and characterize eleven lactate-inducible systems from *Escherichia coli*, *Cupriavidus necator*, and *Pseudomonas* spp. The specificity and dynamics of these systems in response to L- and D-lactate, or structurally similar compounds are investigated. We demonstrate that the inducible systems *Ec*LldR/P_*lldP*_ and *Cn*GntR/P_*H16_RS19190*_ respond only to the L-lactate, exhibiting approximately 19- and 24-fold induction, respectively. Despite neither of the examined bacteria possess the D-lactate-specific inducible system, the *Pa*PdhR/P_*lldP*_ and *Pf*PdhR/P_*lldP*_ are induced approximately 37- and 366-fold, respectively, by D-lactate and can be used for developing biosensor with improved specificity. The findings of this study provide an insight into understanding of L- and D-lactate-inducible systems that can be employed as sensing and tuneable devices in synthetic biology.

## Introduction

Optically pure L- and D-lactic acids are used for synthesis of bioplastic PLA^1^ that has shown potential to replace traditional petroleum-based plastics. PLA exhibits physicochemical, mechanical, and thermal properties comparable to industrial plastics and most importantly, it is biodegradable contributing to the reduction of environmental pollution and plastic recycling costs^2^. Naturally, L- or D-lactic acid can be produced by lactic acid bacteria, some *Bacillus* species and filamentous fungi at close to 100% theoretical yields^3^. Seeking to reduce the expenditure of fermentation and substrate-associated costs, genetically modified strains are increasingly being developed for utilization of alternative carbon sources^4^.

Bacteria can not only produce lactic acid but also utilise it as a carbon source, typically involving two types of lactate dehydrogenases, NAD-dependent lactate dehydrogenases (nLDHs) and NAD-independent lactate dehydrogenases (iLDHs). nLDH catalyses the oxidation of lactate to pyruvate by using NADH as electron acceptor, whereas iLDH uses a flavin-dependent mechanism^5^. iLDHs are classified into NAD-independent L-lactate dehydrogenase (L-iLDH) and NAD-independent D-lactate dehydrogenase (D-iLDH) according to the substrate specificity. Most of the L-iLDHs belong to α-hydroxy acid oxidizing flavoproteins family and use flavin mononucleotide (FMN) as a cofactor. These dehydrogenases can be divided into three groups by the electron acceptor, which can utilise quinones, oxygen or cytochrome *c*^5^. D-iLDHs potential grouping is similar. Most bacterial D-iLDHs use flavin adenine dinucleotide (FAD) as a cofactor and belong to FAD-binding 4 family. According to the electron acceptor, LDH members of this family are further divided into quinone- or cytochrome *c*-dependent D-iLDH, and Fe-S D-iLDH^5,6^.

Genes encoding for L-iLDH, D-iLDH and lactate permease often form lactate utilization operon, the expression of which is regulated by a transcription factor. Such lactate catabolism-related genes and their corresponding transcriptional regulators (TRs) have been identified previously in several classes of bacteria including *Gammaproteobacteria*^7–9^, *Actinobacteria*^10^, *Bacilli*^11,12^, *Deltaproteobacteria*^13,14^, and *Clostridia*^15^. A majority of the lactate utilization operons reported so far are controlled by the GntR family TRs with one exception of LysR family regulator in *Shewanella oneidensis*^16^.

Inducible gene expression systems, composed of inducer-responsive TR and associated genetic elements including promoter and operator sequences, have increasingly been used for developing genetically encoded biosensors. Some inducible systems respond to distinct chemical ligands and they can be used for real-time monitoring of these compounds extracellularly and intracellularly, including applications such as a high-throughput screening of mutant libraries, dynamic pathway control, and adaptive laboratory evolution^17,18^. For L-lactate, an inducible gene expression system from *E. coli* has been previously applied for monitoring mammalian cell cultures^19^. Recently, an inducible system exhibiting sixfold higher induction by D-lactate than that by L-lactate has been reported in *Pseudomonas fluorescens*^9^.

Here, we report the identification, characterization, and specificity of L- and D-lactate inducible-gene expression systems from gammaproteobacteria *E. coli* MG1655 and *Pseudomonas* spp., and betaproteobacterium *C. necator* H16. To identify lactate-responsive-inducible systems, the L- and D-lactate catabolism-related gene clusters were analysed using a generalised genome-wide approach described previously^20^. The putative lactate-inducible systems are investigated for their response to L- and D-lactate, as well as structurally similar glycolate, glyoxylate, 3-hydroxypropionate and pyruvate, followed by a comprehensive characterization and specificity evaluation. Furthermore, to expedite synthetic biology and metabolic engineering efforts, the dynamics of inducible systems is parameterized and their suitability for controlling orthogonal gene expression is demonstrated.

## Results

### Identification of putative lactate-inducible systems in *E. coli* MG1655, *C. necator* H16, and *Pseudomonas* spp

**Figure 1:**
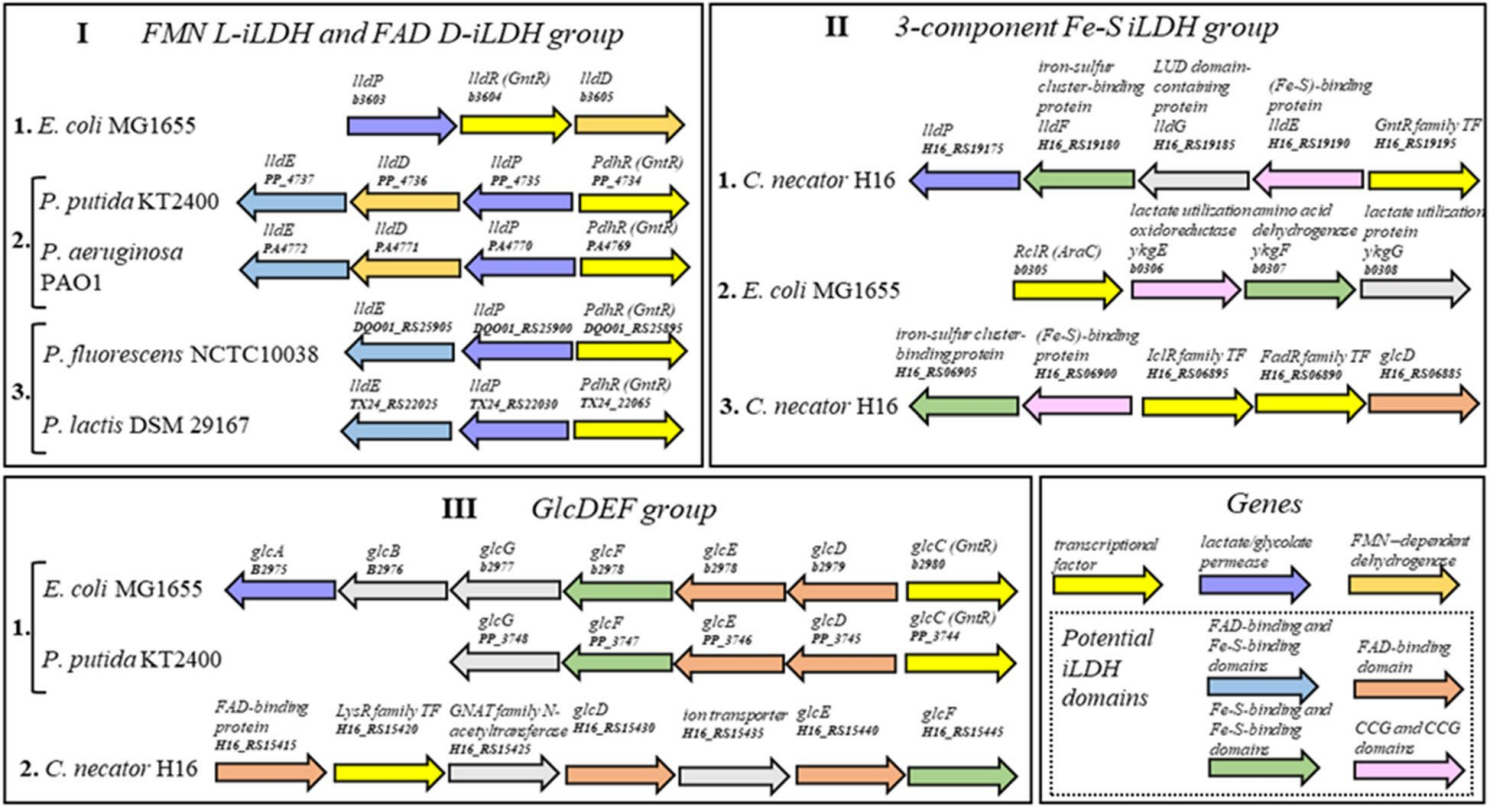
Genomic organization of gene clusters containing lactate catabolism-related operons and associated TR genes in *E. coli* MG1665, *C. necator* H16 and *Pseudomonas* spp. Gene clusters are divided into groups I, II and III as described Results section. Only gene clusters containing at least two lactate catabolism-associated genes and adjacent TR gene are included. Locus tags, protein functions, and protein domains are indicated.

Several lactate catabolism-related gene clusters have been previously characterized in *E. coli*, *P. aeruginosa*, and *P. fluorescens*^7–9,21^. To identify putative inducible systems, we searched GenBank (www.ncbi.nlm.nih.gov)^22^ for gene clusters with commonly occurring arrangements, where TR is transcribed in either the same or different orientation of lactate catabolism-related genes and it either forms a part of or is adjacent to such gene cluster. Based on the homology of functional genes encoding LDHs and lactate permeases, we identified potential operons that contained at least two genes related to the lactate catabolism and the adjacent TR gene in *E. coli* MG1655, *C. necator* H16, and *Pseudomonas* spp.. The glycolate metabolism related gene clusters were also considered, as their involvement in the D-lactate catabolism has been reported previously^23^. All gene clusters were grouped according to the following two rules. First, they were divided into groups (I, II and III) according to the type of enzyme, which was determined by the requirement of cofactor and electron acceptor as described above. Second, groups were divided into subgroups (1, 2 and 3) based on the protein homology (with amino acid sequence identity of more than 40%) and the TR family (Fig. 1). Subsequently, identified gene clusters were divided into group I, representing FMN-dependent L-iLDHs (EC 1.1.2.3) and FAD-dependent D-iLDHs (EC 1.1.2.4), group II, containing 3-component Fe-S iLDHs^24^, and group III, associated with the glycolate catabolism.

*C. necator* H16 gene cluster *H16_RS19175-H16_RS19195* was allocated to the group II, despite that the lactate permease (locus tag *H16_RS19175*) exhibits a high homology with 65% amino acid sequence identity to the lldP of *E. coli* MG1655 (locus tag *b3603*) from group I. In this case, other three genes encode proteins with 34%, 40%, and 31% sequences identity to the 3-component Fe-S iLDH encoded by *ykgEFG* operon from *E. coli* (Supplementary Table S1) and 32%, 40%, and 30% amino acid sequences identity to *S. oneidensis* lldEFG^24^.

Another lactate utilization operon identified in *C. necator* H16 is controlled by potential inducible system *Cn*GntR/P_*H16_RS19190*_. Iron-sulfur cluster-binding protein (locus tag *H16_RS19180*) and (Fe-S)-binding protein (locus tag *H16_RS19190*) encoded by genes of this operon exhibit 40% and 40.5% sequence identity, respectively, with the proteins encoded by the gene cluster *H16_RS06895-H16_RS06915* that contains potential inducible system *Cn*IclR/P_*H16_RS06900*_ (Supplementary Table S1). Transcription factors of group II share no significant similarity with each other and belong to different TR families: GntR, AraC, and IclR. Therefore, all three gene clusters of group II were allocated into different subgroups 1, 2 and 3.

Two analogous *E. coli* MG1655 and *P. putida* KT2440 glycolate catabolism-related gene clusters containing GntR family TR were allocated to the subgroup 1 of group III. Although, similar gene cluster with genes *glcD*, *glcE*, *glcF* encoding proteins with greater than 47% sequence identity to *E. coli* MG1655 homologs was also identified in *C. necator* H16 (Supplementary Table S1), the expression of these genes is potentially regulated by the LysR family TR and, therefore this gene cluster was allocated to subgroup 2 of group III.

Altogether, eleven putative lactate catabolism-related gene clusters were identified in *E. coli* MG1655, *C. necator* H16, and *Pseudomonas* spp. Corresponding putative lactate-inducible gene expression systems, composed of TR gene and relevant promoter/operator region, were selected for further characterization.

### Lactate-inducible systems: validation and evaluation

**Figure 2:**
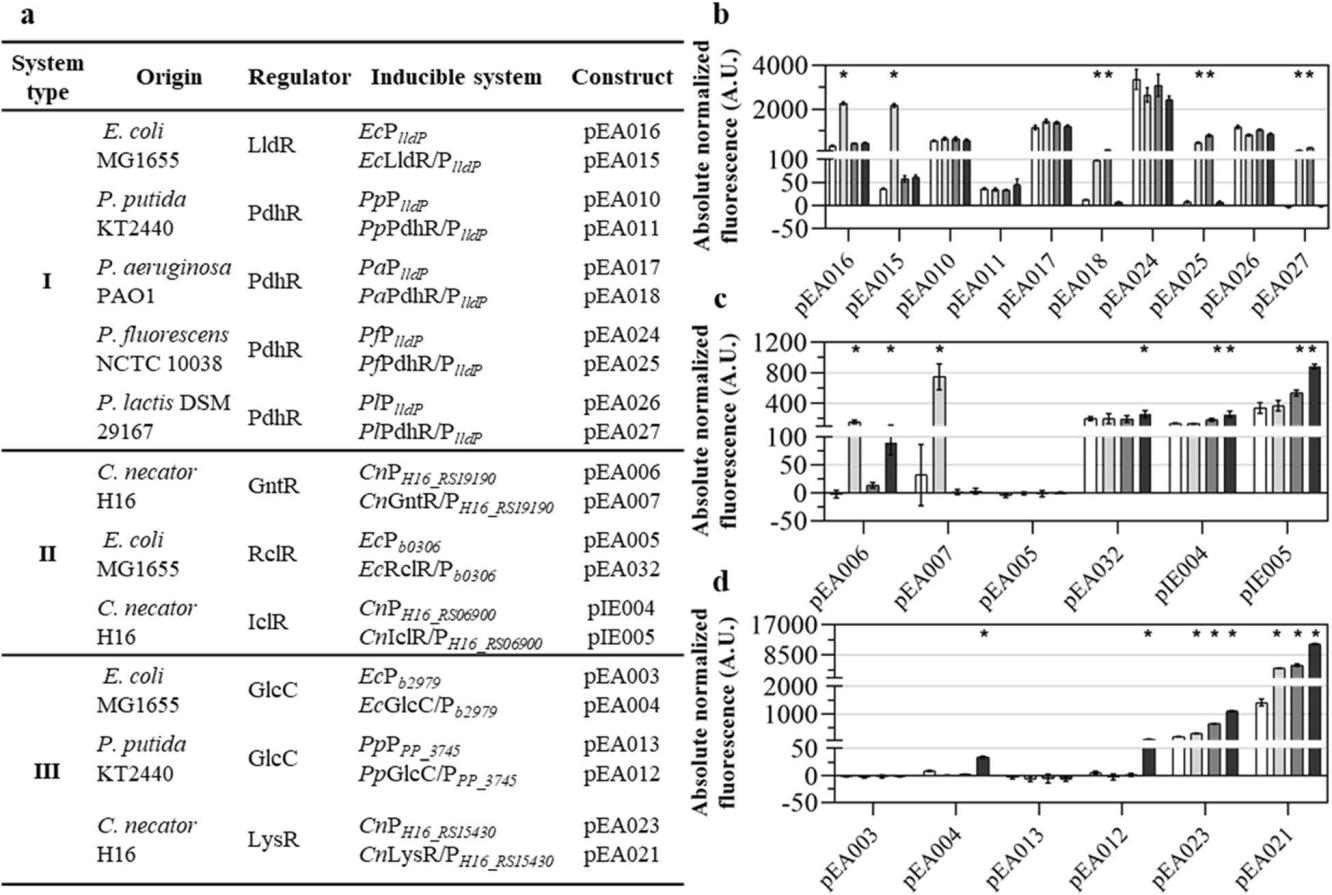
Quantitative evaluation of lactate-inducible systems. (**a**) Summary of the identified inducible systems, including the system type (I, II and III) based on the gene cluster grouping in Fig. 1, system origin, TR name, inducible promoter or system, and corresponding plasmid construct. Schematic illustration of plasmid constructs is provided in the Supplementary Fig. S1. (**b**–**d**) Single time-point RFP fluorescence measurements taken 6 h after addition of inducer for inducible systems type I (**b**), II (**c**), and III (**d**). The fluorescence output was determined in the absence of inducer (white) and in the presence of either L-lactate (light grey), D-lactate (dark grey), or glycolate (black) to a final concentration of 5 mM. Inducible systems from *E. coli* and *C. necator* were assayed using *E. coli* DH5α and *C. necator* H16, respectively, as hosts, whereas systems from *Pseudomonas* spp. were tested in *P. putida* KT2440. Cells were grown in minimal medium containing 0.4% glucose (in the case of *C. necator* H16 supplemented with 0.4% gluconate). Error bars represent standard deviations of three biological replicates,*p < 0.001, unpaired two-tailed *t*-test.

In order to examine the response of putative lactate-inducible systems to the L- and D-lactate or structurally similar compounds such as glycolate, glyoxylate, 3-hydroxypropionate and pyruvate, two types of plasmid constructs were assembled: the first carrying inducible promoter only and the second carrying inducible promoter and TR gene (Fig. 2a and Supplementary Information). To enable the measurement of promoter activity, both types of constructs include mRFP reporter gene located downstream to the inducible promoter. In the second type of construct, the TR gene is positioned in the opposite direction to the inducible promoter and reporter gene. *E. coli* DH5α, *C. necator* H16, and *P. putida* KT2440 strains transformed with these constructs were used as whole-cell biosensors to evaluate the system’s response to different ligands. Logarithmically growing cells in minimal medium were used to measure the RFP fluorescence output 6 h after addition of the inducer (Fig. 2b–d) ensuring that the gene expression is close to steady-state.

Amongst eleven putative lactate-inducible systems, two L-lactate specific systems were identified with *Ec*LldR/P_*lldP*_ characterized previously^19,25,26^. The *Ec*LldR/P_*lldP*_ (plasmid pEA015) exhibited approximately a 19-fold dynamic range with 5 mM L-lactate (Table 1 and Fig. 2b), complementing results reported in^19^.

**Table 1:** Parameters of the lactate-inducible systems.

Construct (inducible system)	Application host	Inducer	Dynamic range, in -fold	*K*_*m*_ (mM)	Hill coefficient
pEA015 (*Ec*LldR/P_*lldP*_)	*E. coli* DH5α	L-lactate	19.0 ± 2.3^a^	0.22 ± 0.05	1.18 ± 0.06
pEA018 (*Pa*PdhR/P_*lldP*_)	*P. putida* KT2440	L-lactate	43.9 ± 6.7^a^	0.04 ± 0.01	1.16 ± 0.08
		D-lactate	37.4 ± 0.1^a^	0.07 ± 0.02	0.91 ± 0.04
pEA025 (*Pf*PdhR/P_*lldP*_)	*P. putida* KT2440	L-lactate	672.1 ± 122.2^a^	0.06 ± 0.02	1.06 ± 0.21
		D-lactate	365.9 ± 139.5^a^	0.08 ± 0.01	1.05 ± 0.21
pEA027 (*Pl*PdhR/P_*lldP*_)	*P. putida* KT2440	L-lactate	42.8 ± 9.4^b^	ND	ND
		D-lactate	78.3 ± 11.9^b^	ND	ND
pEA007 (*Cn*GntR/P_*H16_RS19190*_)	*C. necator* H16	L-lactate	23.7 ± 4.9^a^	0.67 ± 0.21	1.19 ± 0.10
pIE005 (*Cn*IclR/P_*H16_RS06900*_)	*C. necator* H16	glycolate	2.6 ± 0.5^b^	ND	ND
pEA021 (*Cn*LysR/P_*H16_RS15430*_)	*C. necator* H16	D-lactate	3.9 ± 0.6^b^	ND	ND
		glycolate	7.6 ± 0.8^b^	ND	ND

Data are mean ± SD, n = 3. ND-not determined.

^a^calculated according to Methods section, formula (3).

^b^calculated by dividing the maximum level of fluorescence output by the basal level of fluorescence output.

For gene clusters of 3-component iLDH (group II; Fig. 1), putative inducible systems *Cn*GntR/P_*H16_RS19190*_ (pEA007), *Ec*RclR/P_*b0306*_ (pEA032), and *Cn*IclR/P_*H16_RS06900*_ (pIE005) were proposed. Different type of TR was identified for each of three gene clusters in this group. Moreover, the 3-component iLDH proteins exhibit less than 40% identity between homologs encoded by gene clusters in group II, indicating a high degree of genetic diversification. This implicates that different mechanisms have likely evolved for the gene expression regulation of 3-component iLDH operons (Fig. 1). The *Cn*GntR/P_*H16_RS19190*_ was induced approximately 24-fold by L-lactate (Table 1 and Fig. 2c). It appears to be controlled by the GntR family TR evidently involved in the L-lactate catabolism-related gene expression regulation (Supplementary Fig. S2). Whereas *Ec*RclR/P_*b0306*_ or *Cn*IclR/P_*H16_RS06900*_ showed only a minor response to the glycolate of approximately twofold or D-lactate and glycolate of approximately 2- and threefold, respectively (Fig. 2c). Intriguingly, RclR is a redox-regulated transcription factor of the AraC family, which activates the expression of genes required for survival of reactive chlorine stress^27^. Besides, the expression from promoter P_*b0306*_ is subjected to the carbon catabolic repression in the presence of glucose and it is derepressed when cells are grown using L- and D-lactate as a carbon source (pEA032; Fig. 2c and Supplementary Fig. S3).

Second *C. necator* H16 gene cluster, allocated to the group II and containing genes encoding 3-component iLDH, revealed putative inducible system *Cn*IclR/P_*H16_RS06900*_ with atypical transcription factor IclR (Fig. 1). *C. necator* H16 cells harbouring plasmid pIE005 with promoter and TR gene (*Cn*IclR/P_*H16_RS06900*_) showed marginally higher induction level of approximately 2- and threefold with D-lactate and glycolate, respectively, than those harbouring pIE004 with promoter only (*Cn*P_*H16_RS06900*_) (Fig. 2a,c). These results suggest that either the IclR has minor effect on the activation of promoter *Cn*P_*H16_RS06900*_ or the expression of *iclR* gene is suppressed under tested conditions. To investigate this further, the *iclR* gene was placed under control of either a strong constitutive synthetic promoter P_*13*_^28^ or inducible system *Ec*AraC/P_*araBAD*_, resulting in the construct pEA014 or pEA028, respectively. The comparison of absolute normalized fluorescence outputs, obtained using pIE005, pEA014 and pEA028, showed that the placing of *iclR* gene under the control of either constitutive or inducible promoter significantly affects the activity of *Cn*P_*H16_RS06900*_. However, the IclR has little effect on the inducible system’s response to D-lactate and glycolate (Supplementary Figs. S4 and S5).

Subsequently, we hypothesized that *Cn*P_*H16_RS06900*_ could be regulated by unknown transcription factor with one specific candidate identified as FadR encoded by a gene located downstream to the *iclR*. We constructed two additional plasmids pEA019 and pEA020 containing *fadR* gene (Supplementary Fig. S4a). However, *C. necator* H16 cells harbouring plasmids pEA019 and pEA020 exhibited fluorescence outputs similar to those observed with pIE005 and pIE004 (Supplementary Fig. S4b and Fig. 2c). Furthermore, the comparison of absolute normalized fluorescence outputs, obtained using pIE005, pEA019 and pEA020 in *E. coli* DH5α, showed no response to either D-lactate or glycolate (Supplementary Fig. S6). Above results suggest that it is unlikely that FadR regulates the *Cn*P_*H16_RS06900*_ and further research is required to elucidate the roles of IclR and FadR in the gene expression regulation of *H16_RS06900-H16_RS06905* cluster.

Of gene clusters allocated to the group III (Fig. 1), inducible systems *Ec*GlcC/P_*b2979*_ (pEA004) and *Pp*GlcC/P_*PP_3745*_ (pEA012) were induced approximately 4- and 15-fold, respectively, by glycolate (Fig. 2d). In order to elucidate the regulation of glycolate-inducible system from *C. necator* H16 (Fig. 2a; *Cn*LysR/P_*H16_RS15430*_), the P_*H16_RS15415*_ and P_*H16_RS15430*_ promoter regions (pEA022 and pEA023, respectively), and LysR TR gene with either P_*H16_RS15415*_ (pEA021) or P_*H16_RS15430*_ (pEA030) promoter regions were subjected to evaluation using the reporter system (Supplementary Fig. S7). Absolute normalized fluorescence results showed that P_*H16_RS15430*_ (pEA030) is induced not only by glycolate (approximately sixfold) but also by L- and D-lactate (approximately 2- and fourfold, respectively). A slightly higher induction of approximately 8-, 3- and fourfold by glycolate, L- and D-lactate, respectively, was observed using *Cn*LysR/P_*H16_RS15430*_ (pEA021).

No D-lactate specific inducible system was identified in either *E. coli* MG1655, *C. necator* H16 or *Pseudomonas* spp.. The inducible system *Pf*PdhR/P_*lldP*_ (pEA025), exhibiting the highest dynamic range of approximately 366-fold in response to D-lactate, was also induced approximately 672-fold by L-lactate (Table 1). Similarly, *Pl*PdhR/P_*lldP*_ (pEA027) and *Pa*PdhR/P_*lldP*_ (pEA018) were also induced by both L-lactate (approximately 78- and 44-fold, respectively) and D-lactate (43- and 37-fold, respectively). TRs of these systems act as repressors and the gene expression here is activated in the presence of either L- or D-lactate. Intriguingly, despite exhibiting a high level of homology to the inducible system *Pa*PdhR/P_*lldP*_, the *Pp*P_*lldP*_ (pEA010) and *Pp*PdhR/P_*lldP*_ (pEA011) showed no response to either L- or D-lactate when *P. putida* KT2440 strains harbouring plasmids pEA010 and pEA011 were grown in minimal medium with glucose as a carbon source (Fig. 2b). Further research is required to examine.

### Parameterisation of lactate-inducible systems

**Figure 3:**
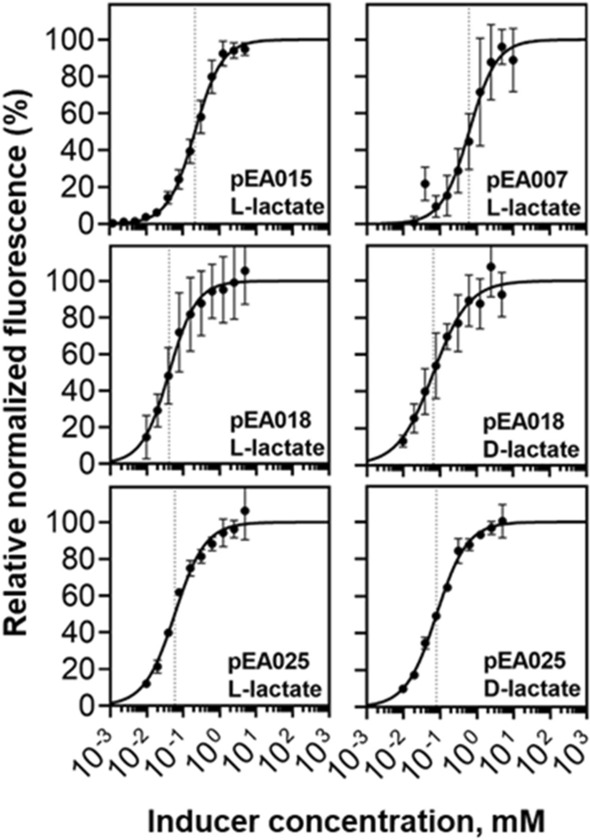
Dose–response curves of L- and D-lactate inducible systems. Relative normalized fluorescence of *E. coli* DH5*α*, *C. necator* H16 and *P. putida* KT2440 harbouring pEA015 with *Ec*LldR*/*P_*lldP*_, pEA007 with *Cn*GntR*/*P_*H16_RS19190*_, and pEA018 with *Pa*PdhR/P_*lldP*_, and pEA025 with *Pf*PdhR/P_*lldP*_ determined in response to different concentrations of either L- and D-lactate 6 h after inducer addition. Level range of L- or D-lactate varied from 0 to 5 mM (0, 0.0003, 0.0006, 0.0012, 0.0024, 0.0048, 0.009, 0.019, 0.039, 0.078, 0.156, 0.313, 0.625, 1.25, 2.5, and 5 mM). The dose–response curves were fitted using Hill function as described in Methods. The maximum level of reporter output *b*_max_ was set to 100%. *K*_m_ is marked using a dotted line. Cells were grown in minimal medium containing 0.4% glucose (in the case of *C. necator* H16 supplemented with 0.4% gluconate). Error bars represent standard deviations of three biological replicates. Source data are provided in Supplementary Data file.

To obtain data of induction kinetics, the fluorescence and absorbance were monitored over time using plasmid-transformed *E. coli* (pEA015), *C. necator* (pEA007), and *P. putida* (pEA018, pEA025, pEA027) carrying inducible systems *Ec*LldR/P_*lldP*_, *Cn*GntR/P_*H16_RS19190*_, *Pa*PdhR/P_*lldP*_, *Pf*PdhR/P_*lldP*_, and *Pl*PdhR/P_*lldP*_, respectively (Supplementary Figs. S8 and S9, respectively). Cells were grown in minimal medium containing 0.4% glucose or gluconate (in the case of *C. necator* H16) and supplemented with L- or D-lactate to a final concentration of 5 mM. Furthermore, L-lactate specific inducible systems *Ec*LldR/P_*lldP*_ (pEA015) and *Cn*GntR/P_*H16_*RS19190_ (pEA007) along with systems *Pa*PdhR/P_*lldP*_ (pEA018) and *Pf*PdhR/P_*lldP*_ (pEA025), responding to both L- and D-lactate, were subsequently evaluated for their dose–response using different concentrations of inducer (from 0 to 5 mM) (Fig. 3). A maximum concentration of 5 mM was used to avoid growth inhibition observed at higher concentrations of lactate. Generally, at this concentration all inducible systems were fully saturated. The parameters of lactate-inducible systems that respond to L-lactate or both lactic acid enantiomers are shown in Table 1. Both L-lactate specific inducible systems *Ec*LldR/P_*lldP*_ (pEA015) and *Cn*GntR/P_*H16_RS19190*_ (pEA007) exhibit *K*_*m*_ values slightly below of 1 mM, indicating that these systems respond to µM–mM concentration range of L-lactate. The *Ec*LldR-regulated gene expression can be tuned over a slightly lower range of approximately 9 µM–1 mM than that of the *Cn*GntR (approximately 80 µM–5 mM). Dose response analysis showed that inducible systems *Pa*PdhR/P_*lldP*_ (pEA018) and *Pf*PdhR/P_*lldP*_ (pEA025) exhibit similar affinities to both lactic acid enantiomers. The gene expression using these constructs can be tuned in the range of approximately 9 µM–2 mM of L- or D-lactate.

### Determination of inducible systems specificity

**Figure 4:**
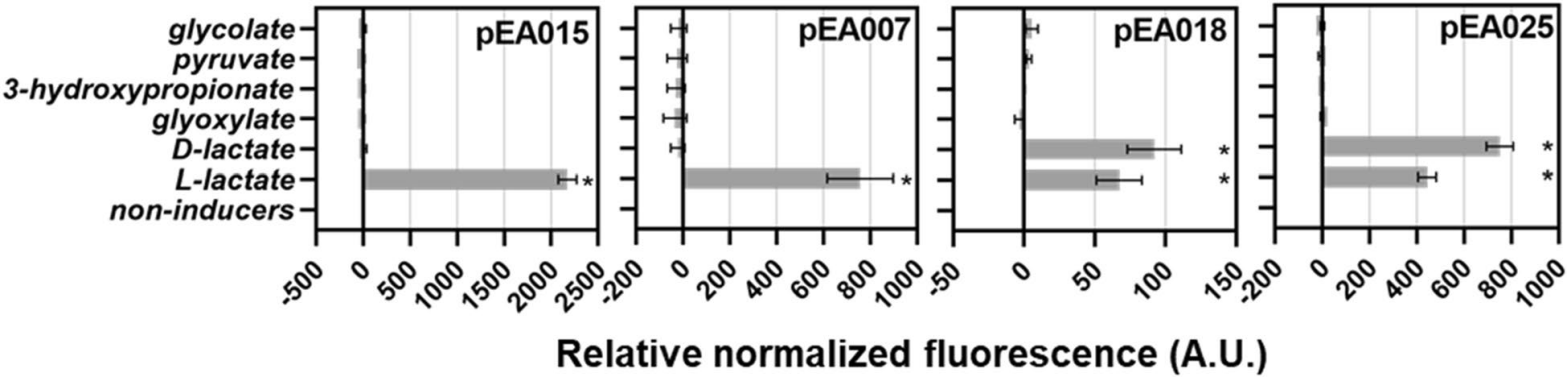
Relative normalized fluorescence of *E. coli* DH5α, *C. necator* H16, and *P. putida* KT2440 harbouring pEA015 with *Ec*LldR/P_*lldP*_, pEA007 with *Cn*GntR/P_*H16_RS19190*_, pEA018 with *Pa*PdhR/P_*lldP*_, and pEA025 with *Pf*PdhR/P_*lldP*_. Single time-point fluorescence measurements were taken 6 h after addition of different compounds to a final concentration of 5 mM. Error bars represent standard deviations of three biological replicates, *p ≤ 0.01 (unpaired *t*-test).

Determination of inducible system specificity can provide a better understanding about the affinity of TR to the lactate or structurally similar compounds, the structural properties required for transcription factor binding, and may allow the list of analogous metabolites to be expanded^29^. Therefore, the response of selected inducible gene expression systems including *Ec*LldR/P_*lldP*_ (pEA015), *Cn*GntR/P_*H16_RS19190*_ (pEA007), *Pa*PdhR/P_*lldP*_ (pEA018), and *Pf*PdhR/P_*lldP*_ (pEA025) to glyoxylate, 3-hydroxypropionate, and pyruvate in addition to glycolate, L- and D-lactate was investigated. Each inductor was added to a final concentration of 5 mM and fluorescence outputs were monitored over time of logarithmically growing cultures in minimal medium containing 0.4% glucose or gluconate (in the case of *C. necator* H16). The results showed that structurally similar compounds, such as glyoxylate, 3-hydroxypropionate, and pyruvate do not induce the tested systems. Inducible systems *Ec*LldR/P_*lldP*_ (pEA015) and *Cn*GntR/P_*H16_RS19190*_ (pEA007) are specific only to the L-lactate, whereas the *Pa*PdhR/P_*lldP*_ (pEA018) and *Pf*PdhR/P_*lldP*_ (pEA025) do not differentiate between L and D enantiomers and respond to both optical forms of lactate (Fig. 4).

### Improvement of the L-lactate-inducible system

**Figure 5:**
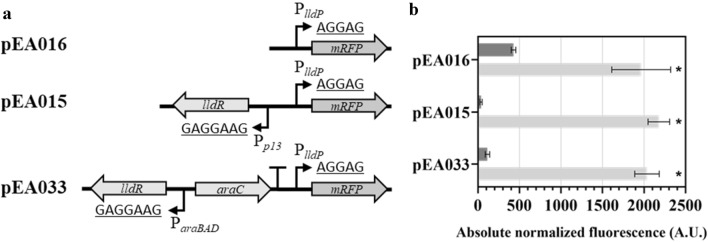
Engineering the L-lactate-inducible system. (**a**) Schematic illustration of the different versions of the *Ec*LldR/P_*lldP*_-inducible system with their corresponding plasmid identifiers and the Shine-Dalgarno sequences are underlined. (**b**) Absolute normalized fluorescence of *E. coli* DH5α carrying reporter constructs with different versions of the *Ec*LldR/P_*lldP*_-inducible system in the absence (dark grey) and presence (light grey) of 5 mM L-lactate (pEA016 and pEA015). In the case of pEA033, the culture was also supplemented with 0.2% L-arabinose. Single time-point RFP fluorescence measurements were taken 6 h after addition of inducer. Cells were grown in minimal medium containing 0.4% glucose. Error bars represent standard deviations of three biological replicates, *p ≤ 0.01 (unpaired *t*-test).

In order to establish if the inducible system *Ec*LldR/P_*lldP*_ can be improved by altering *lldR* expression, constructs containing an intergenic region with promoter P_*lldP*_ and the gene of the transcriptional regulator (*lldR*) under control of either the synthetic promoter P_*13*_ (pEA015) or arabinose inducible system *Ec*AraC/P_*araBAD*_ (pEA033), were evaluated (Fig. 5). Construct pEA016 containing only the intergenic region with promoter P_*lldP*_ was used as a negative control. Single time-point fluorescence measurements of *E. coli* harbouring pEA015, pEA033, and pEA016 were performed in the absence and presence of L-lactate. Results showed that 6 h after supplementation with 5 mM L-lactate and 0.2% L-arabinose (in the case of pEA033) the absolute normalized fluorescence was very similar for all three constructs, whereas the overexpression of *lldR* (pEA015 and pEA033) resulted in reduced fluorescence when L-lactate was absent and pEA015 exhibited almost a complete inhibition. Further analysis revealed that the gene expression was induced by approximately 59-fold for pEA015 and 18-fold for pEA033 (Fig. 5b). This suggests that the promoter P_*13*_ likely enhances the *lldR* expression more than the *Ec*AraC/P_*araBAD*_ and the increase in the LldR amount can stimulate repression of the *Ec*LldR/P_*lldP*_ in the absence of L-lactate.

Simultaneously, to improve inducible system *Ec*LldR/P_*lldP*_, the application of different strength RBSs controlling the LldR synthesis was investigated in the construct pEA015 containing TR gene under control of constitutive promoter P_*13*_. Theoretical translation rates of RBS variants were estimated using an RBS calculator (https://salislab.net/software/)^30^. Absolute normalized fluorescence results obtained by using RBS variants showed that the sequences with different theoretical translation rates exhibit a statistically significant effect on the gene expression and *Ec*LldR/P_*lldP*_ induction (Supplementary Fig. S10). A much improved dynamic range of approximately 100- and 122-fold was achieved when RBSs with theoretical translation rates of 179,032.18 and 51,067.85 A.U., respectively, were introduced into pEA015 comparing to approximately a 59-fold induction obtained using RBS with a theoretical translation rate of 19,143.57 A.U.. Altogether, these results show that the inducible system *Ec*LldR/P_*lldP*_ can be improved by fine-tuning the *lldR* expression.

### Applicability of inducible systems in non-host microorganisms

**Figure 6:**
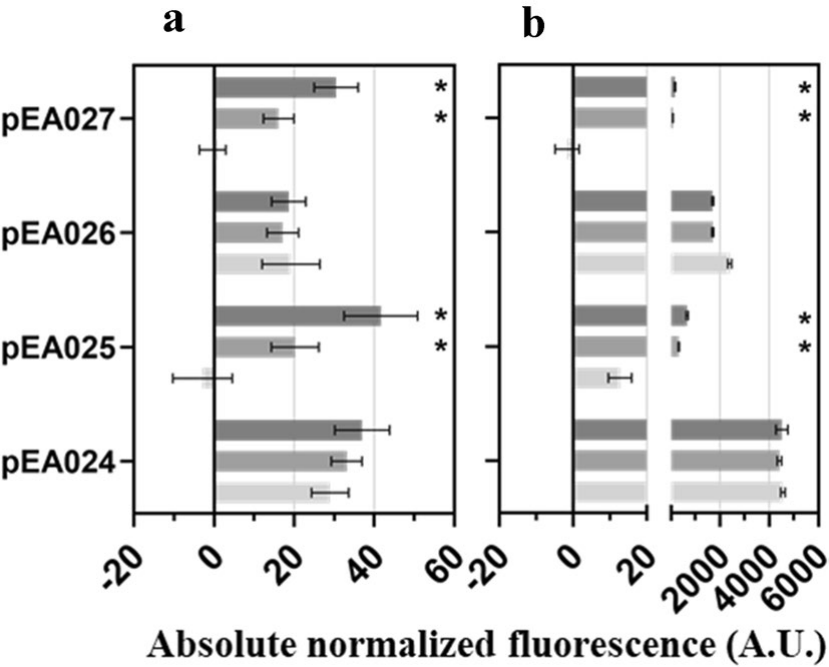
Inducible systems *Pf*PdhR/P_*lldP*_ and *Pl*PdhR/P_*lldP*_ from *P. fluorescens* NCTC 10,038 and *P. lactis* DSM 29,167, respectively, mediate controllable gene expression in *C. necator* H16 and *P. putida* KT2440. Single time-point fluorescence measurements of (**a**) *C. necator* H16 and (**b**) *P. putida* KT2440 carrying the plasmids pEA024 and pEA026 containing ‘promoter only’ (*Pf*PdhR/P_*lldP*_ and *Pl*PdhR/P_*lldP*_, respectively), and plasmids pEA025 and pEA027 containing TR gene and promoter (*Pf*PdhR/P_*lldP*_ and *Pl*PdhR/P_*lldP*_, respectively). RFP fluorescence output was determined in the absence of inducer (light grey) and 12 h after extracellular supplementation with L-lactate (middle grey) and D-lactate (dark grey) to a final concentration of 5 mM. Cells were grown in minimal medium containing 0.4% glucose (in the case of *C. necator* H16 supplemented with 0.4% gluconate). Error bars represent standard deviations of three biological replicates, *p ≤ 0.01 (unpaired *t*-test).

To our knowledge, the lactate-inducible gene expression systems have not been previously studied in non-host organisms^9,19^. To evaluate if the inducible systems identified in this study can be used in other host microorganisms, most prominent systems such as *Ec*LldR/P_*lldP*_, *Pf*PdhR/P_*lldP*_ and *Pl*PdhR/P_*lldP*_ were tested in betaproteobacterium *C. necator* H16 and gammaprotebacterium *P. putida* KT2440. First, the inducible system *Ec*LldR/P_*lldP*_ was introduced into these strains on the plasmids pEA015 and pEA033. No significant change in fluorescence output was observed in the presence of L-lactate, when plasmid-transformed cells were grown in minimal medium with 0.4% glucose (*P. putida*) or gluconate (*C. necator*) as carbon source (Supplementary Fig. S11 for pEA015 and Supplementary Fig. S12a for pEA033). However, when *C. necator* cells, harbouring pEA033 with *lldR* under control of the *Ec*AraC/P_*araBAD*_, were grown using L-lactate as sole carbon source, approximately a 58-fold induction was observed (Supplementary Fig. S12). These results shows that in *C. necator* H16, when *lldR* is under control of arabinose-inducible system (pEA033), the *Ec*LldR/P_*lldP*_ is activated in the presence of L-lactate as sole carbon source and it seemingly overcomes a threshold associated with the carbon catabolite repression^31^. Our results complement the recently published study that analyses the catabolic repression of the *lldPDE* promoter from *E. coli* MG1655 in the presence of glucose^26^. Second, the inducible systems *Pf*PdhR/P_*lldP*_ (pEA025) and *Pl*PdhR/P_*lldP*_ (pEA027), induced by both lactic acid enantiomers in *P. putida* KT2440 (Fig. 2a), were investigated using *C. necator* H16 as a host (Supplementary Figs. S13 and S14). RFP fluorescence outputs revealed that the *Pf*PdhR/P_*lldP*_ and *Pl*PdhR/P_*lldP*_ are induced approximately 7- and sixfold, respectively, by L-lactate and 14- and 12-fold, respectively, by D-lactate (Fig. 6).

## Discussion

The replacement of chemical synthesis by renewable bio-based processes is becoming a major international objective aimed at the reducing reliance on fossil fuels and energy. The lactic acid is an important platform chemical with multiple applications including synthesis of biodegradable PLAs. In the last decade, substantial biotechnology efforts have been made to identify and develop new microbial strains suitable for the bio-based production of L- and D-lactate^3^. The lactic acid was ranked as top one bio-based chemical opportunities in the UK, due to the large market potential and environmental concerns related to the use of fossil-derived and non-renewable plastics^32^.

TF-based inducible gene expression systems are useful tools for screening and development microbial strains with improved production of chemical compounds including lactic acid. Inducible systems have been widely used in the synthetic biology and biotechnology, helping to the development of improved biosynthesis processes and due to their adaptability to design–build–test-learn cycle^17,33,34^. In this study, we identified eleven putative gene clusters related to the lactate catabolism and pursued characterization of corresponding inducible gene expression systems from *E. coli* MG1655, *C. necator* H16, and *Pseudomonas* spp.. Amongst these, two inducible systems specific to the L-lactate were characterized. The *Ec*LldR/P_*lldP*_ (pEA015) dynamic range of approximately 19-fold was in agreement with an 18.63-fold induction observed using the same P_*lldP*_ promoter and LldR TR from *E. coli* in the presence of 14 mM L-lactate^19^.

All inducible systems from lactate catabolism-related gene clusters in group I (Fig. 1) are regulated by TRs (LldR and PdhR) that belong to the FadR subfamily of the GntR family. Members of same subfamily control inducible systems *Cn*GntR/P_*H16_RS19190*_ (pEA007), *Ec*GlcC/P_*b2979*_ (pEA004), and *Pp*GlcC/P_*PP_3745*_ (pEA012) of groups II and III. The FadR subfamily is a diverse group of TRs that regulate various metabolic processes in bacteria by binding organic ligands, mostly carboxylic acids, and then undergoing conformational changes affecting the DNA-binding. Members of this family of TRs are involved in the regulation of various central metabolism-related pathways including gluconate (GntR), galactonate (DgoR), glycolate (GlcC), lactate (LldR), and pyruvate (PdhR)^35,36^. In the case of gluconate catabolism, GntR acts as a transcriptional repressor and the *gnt* gene cluster expression is derepressed in the presence of gluconate that interferes with the TR binding to the promoter, as first reported in *Bacillus subtilis*^37^. Moreover, *gnt* operon expression can be regulated by the catabolite control protein (CcpA) through the carbon catabolite repression and activated by the cAMP receptor protein (CRP)-cAMP complex^38^. Similarly, our data demonstrate that the expression of lactate catabolism-related operons in group I are regulated by the L- and D- lactate and carbon catabolite repression.

Inducible systems found to be regulated by FadR subfamily TRs and responding to the L- or D-lactate were characterized for their specificity and orthogonality in different bacterial species. Our results demonstrated that the selected inducible systems *Ec*LldR/P_*lldP*_ (pEA015), *Cn*GntR/P_*H16_RS19190*_ (pEA007)*, Pa*PdhR/P_*lldP*_ (pEA018), and *Pf*PdhR/P_*lldP*_ (pEA025) exhibit a high specificity toward L-lactate or both lactic acid enantiomers. Moreover, *Pa*PdhR/P_*lldP*_ (pEA018) and *Pf*PdhR/P_*lldP*_ (pEA025) can function and are induced by L- and D-lactate in non-host organisms such as the betaproteobacterium *C. necator* H16. This suggests that these inducible systems can be used to orthogonally regulate the gene expression of biosynthetic pathways or other genetic circuits in the wider host range.

Although a few systems, inducible by both L- and D-lactate, were identified in *Pseudomonas* spp., neither of eleven inducible systems subjected to the investigation was found to respond to the D-lactate solely. Contrary to a recent report by Singh et al*.*^9^, our data showed that the inducible system *Pf*PdhR/P_*lldP*_ (pEA025) responds to both L- and D-lactic acid enantiomers to a similar degree. Therefore, further work is required to identify and develop the D-lactate specific inducible system. We propose that by applying directed-evolution and mutagenesis strategies PdhR TR from *Pseudomonas* spp. can be engineered to specifically recognize and drive gene expression in response to the D-lactate.

## Methods

### Gene cluster identification in bacterial genomes

A search for putative gene clusters responsible for L- and/or D-lactate catabolism was performed in the GenBank database (https://www.ncbi.nlm.nih.gov/)^22^. Protein homologs encoded by genes in these clusters were identified and homology determined using a basic local alignment search tool (BLAST) (https://blast.ncbi.nlm.nih.gov/Blast.cgi). The primary search in genomes of *E. coli* MG1655, *C. necator* H16, and *Pseudomonas* spp. was performed using sequences of proteins encoded by *lldD* and *lldP* genes from *E. coli* MG1655 (Supplementary Materials and Methods). Secondary searches in genomes of *E. coli* MG1655, *C. necator* H16, and *Pseudomonas* spp. were carried out using sequences of proteins encoded by genes found to be adjacent to the genes exhibiting identity to the *lldD* and *lldP* of *E. coli* MG1655 and located in the clusters associated with lactate catabolism.

### Chemicals

All chemicals used as inducers in this study are listed in Supplementary Table S2.

### Base strains and media

All strains used in this study are listed in Supplementary Table S3. *E. coli* Top10 and *E. coli* DH5α (Invitrogen, USA) were grown in Luria–Bertani (LB) medium and used for plasmid propagation as described by^39^. For reporter gene assays, *E. coli* DH5α, and *P. putida* KT2440 strains were cultivated at 30 °C in M9 minimal medium supplemented with 1 μg/mL thiamine, 0.4 mM leucine, and 0.4% (w/v) glucose^39^, whereas *C. necator* H16 cells were grown in minimal medium containing 0.4% (w/v) sodium gluconate^40^ at 30 °C. Antibiotics were added to the growth medium at the following concentrations: 25 μg/mL or 50 μg/mL chloramphenicol for *E. coli* or *C. necator* H16, respectively, and 25 μg/mL tetracycline for *P. putida* KT2440.

To estimate catabolic repression, minimal medium was supplemented with 44 mM L- or D-lactate as a carbon source by replacing 0.4% (w/v) glucose.

### Standard DNA techniques

Plasmid DNA was purified by using the GeneJET Plasmid Miniprep Kit (Thermo Scientific, Lithuania). Microbial genomic DNA was extracted employing the GenElute Bacterial Genomic DNA Kit (Sigma, USA). The Zymoclean Gel DNA Recovery Kit (Zymo, USA) was employed to extract gel-purified linearised DNA. Phusion High-Fidelity DNA polymerase, restriction enzymes and T4 DNA Ligase were purchased from Thermo Scientific (Lithuania) and reactions were set up according to the manufacturer’s protocol. NEBuilder HiFi DNA assembly kit was purchased from New England Biolabs (NEB, USA) and reactions were performed according to the manufacturer’s recommendations.

Chemical competent cells of *E. coli* were prepared and transformed as described by^39^. Electrocompetent *C. necator* H16 and *P. putida* KT2440 were prepared and transformed as described by^41^.

### Plasmid construction

Oligonucleotide primers were synthesized by Sigma-Aldrich (USA) and Invitrogen (USA) (Supplementary Table S4). Plasmids were constructed by employing either the NEBuilder Hifi DNA assembly method or by restriction enzyme-based cloning procedures. Constructs were verified by DNA sequencing (Eurofins Genomics, Germany). For plasmids transferred to *P. putida* KT2440, the antibiotic resistance gene was changed from chloramphenicol to tetracycline. A detailed construction description for each plasmid is provided in the Supplementary Information and graphic plasmids overview presented in Supplementary Information Fig. S1. The mRFP1 protein was used in all constructed plasmids and its nucleotide and protein sequence is given in Supplementary Information.

### RFP fluorescence assay

RFP fluorescence was measured using an Infinite M200 PRO (Tecan, Austria) microplate reader. The fluorescence bottom-reading mode was applied for RFP excitation and emitted light measurement using 585 and 620 nm wavelengths with 9 and 20 nm bandwidths, respectively. The gain factor was set to 120%. Absorbance was measured at 600 nm wavelength with 9 nm bandwidth. RFP fluorescence and absorbance were quantified over time and their values were corrected for autofluorescence and autoabsorbance of the medium, respectively. As reported previously^29^, the autofluorescence of cells was similar to the fluorescence of the medium not exceeding 1 A.U. and, therefore, it was considered insignificant. An absolute normalized fluorescence (ANF) was calculated as described previously^42^ using formula (1).

1 $$ANF = \frac{{RFP_{raw} - RFP_{medium} }}{{OD_{raw} - OD_{medium} }}$$

The fold induction was calculated by dividing the ANF value of the induced sample by the ANF of the uninduced sample. Relative normalized fluorescence (A.U.) values as shown in Fig. 4 were obtained by subtracting the absolute normalized fluorescence of the uninduced cells from the absolute normalized fluorescence values of cells supplemented with the inducer.

### Parametrization of inducible system

To obtain system parameters, absolute normalized fluorescence values (RFP) were plotted as a function of inducer concentration using software GraphPad Prism 8 and a non-linear least-squares fit was performed using the Hill function (2) as described previously^42^:

2 $$RFP = b_{max} \times \frac{{I^{h} }}{{K_{m}^{h} + I^{h} }} + b_{min}$$

The system parameters were calculated according to this function (2): maximum rate of RFP synthesis and basal level of RFP synthesis (*b*_max_ and *b*_min_, respectively), the concentration of inducer (*I*), the Hill coefficient (*h*), and the inducer concentration that mediates half-maximal reporter output (*K*_m_).

The dynamic range *μ* was calculated using formula (3):

3 $$\mu = \frac{{b_{max} }}{{b_{min} }}$$

Relative normalized fluorescence (%) values as shown in Fig. 3 were obtained calculated using ANF values at a specific inducer concentration in formula (4):

4 $$Relative\ normalized\ fluorescence \left( \% \right) = 100 \times \left( {\frac{{ANF - b_{min} }}{{b_{max} }}} \right)$$

## Supplementary Information

Supplementary Information 1.

Supplementary Information 2.
