# Supplementary material for: Identification and characterization of L- and D-lactate-inducible systems from Escherichia coli MG1655, Cupriavidus necator H16 and Pseudomonas species
Source: Sci Rep. 2022 Feb 8;12:2123. doi: 10.1038/s41598-022-06028-7 (PMC8827060; doi:10.1038/s41598-022-06028-7)
Supplement: Supplementary file 2 — Supplementary Information 2. [file 41598_2022_6028_MOESM2_ESM.pdf]

Supplementary Information

Identification and characterization of L- and D-lactate-inducible systems from *Escherichia coli* MG1655, *Cupriavidus necator* H16 and *Pseudomonas* species

Ernesta Augustiniene[a] and Naglis Malys[a,b]*

[a]Bioprocess Research Centre, Faculty of Chemical Technology, Kaunas University of Technology, Radvilėnų pl. 19, Kaunas, Lithuania

[b]Department of Organic Chemistry, Faculty of Chemical Technology, Kaunas University of Technology, Radvilėnų pl. 19, Kaunas, Lithuania

*Corresponding author, email: naglis.malys@ktu.lt

Supplementary Materials and Methods

Protein sequences used in the sequence similarity search with the BLAST

Protein sequence encoded by *lldD* gene from *E. coli* MG1655:

miisaasdyraaaqrilppflfhymdggayseytlrrnvedlsevalrqrilknmsdlslettlfneklsmpvalapvglcgmyarrgev qaakaadahgipftlstvsvcpieevapaikrpmwfqlyvlrdrgfmrnalerakaagcstlvftvdmptpgaryrdahsgmsgpnaa mrrylqavthpqwawdvglngrphdlgnisaylgkptgledyigwlgnnfdpsiswkdlewirdfwdgpmvikgildpedardav rfgadgivvsnhggrqldgvlssaalpaiadavkgdiailadsgirngldvvrmialgadtvllgraflyalatagqagvanllnliekem kvamtltgaksiseitqdslvqglgkelpaalapmakgnaa

Protein sequence encoded by *lldP* gene from *E. coli* MG1655:

Mnlwqqnydpagniwlssliaslpilffffaliklklkgyvaaswtvaialavallfykmpvanalasvvygffyglwpiawiiiaavfv ykisvktgqfdiirssilsitpdqrlqmlivgfcfgaflegaagfgapvaitaallvglgfkplyaaglclivntapvafgamgipilvagqvt gidsfeigqmvgrqlpfmtiivlfwimaimdgwrgiketwpavvvaggsfaiaqyldiisslvsllcltlflkrwqpvrvfrfgdlgasq vdmtlahtgytagqvlrawtpflfltatvtlwsippfkalfasggalyewvinipvpyldklvarmppvvseatayaavfkfdwfsatgt ailfaallsivwlkmkpsdaistfgstlkelalpiysigmvlafafisnysglsstlalalahtghaftffspflgwlgvfltgsdtssnalfaalq ataaqqigvsdlllvaanttggvtgkmispqsiaiacaavglvgkesdlfrftvkhsliftcivgvittlqayvltwmip

mRFP1

mRFP1 gene nucleotide sequence:

atggcgagtagcgaagacgttatcaaagagttcatgcgtttcaaagttcgtatggaaggttccgttaacggtcacgagttcgaaatcgaaggt gaaggtgaaggtcgtccgtacgaaggtacccagaccgctaaactgaaagttaccaaaggtggtccgctgccgttcgcttgggacatcctgt ccccgcagttccagtacggttccaaagcttacgttaaacacccggctgacatcccggactacctgaaactgtccttcccggaaggtttcaaat gggaacgtgttatgaacttcgaagacggtggtgttgttaccgttacccaggactcctccctgcaagacggtgagttcatctacaaagttaaact gcgtggtaccaacttcccgtccgacggtccggttatgcagaaaaaaaccatgggttgggaagcttccaccgaacgtatgtacccggaagac ggtgctctgaaaggtgaaatcaaaatgcgtctgaaactgaaagacggtggtcactacgacgctgaagttaaaaccacctacatggctaaaa aaccggttcagctgccgggtgcttacaaaaccgacatcaaactggacatcacctcccacaacgaagactacaccatcgttgaacagtacga acgtgctgaaggtcgtcactccaccggtgcttaa

Protein sequence encoded by *mRFP1* gene:

massedvikefmrfkvrmegsvnghefeiegegegrpyegtqtaklkvtkggplpfawdilspqfqygskayvkhpadipdylklsf pegfkwervmnfedggvvtvtqdsslqdgefiykvklrgtnfpsdgpvmqkktmgweastermypedgalkgeikmrlklkdgg hydaevkttymakkpvqlpgayktdiklditshnedytiveqyeraegrhstga

Construction of plasmids

pBRC1 was constructed as described for pEH006 in [1].

pEA003 was constructed by restriction enzyme-based cloning. Oligonucleotide primers EA021 and EA022 were used to amplify the putative glycolic acid-inducible promoter from *E. coli* MG1655 genomic DNA, and cloned into pBRC1 vector by AatII and NdeI restriction sites.

pEA004 was constructed by restriction enzyme-based cloning. Oligonucleotide primers EA021 and EA023 were used to amplify the putative glycolic acid-inducible system from *E. coli* MG1655 genomic DNA, and cloned into pBRC1 by AatII and NdeI restriction sites.

pEA005 was constructed by restriction enzyme-based cloning. Oligonucleotide primers EA024 and EA025 were used to amplify the putative lactate-inducible promoter from *E. coli* MG1655 genomic DNA, and cloned into pBRC1 by AatII and NdeI restriction sites.

pEA006 was constructed by restriction enzyme-based cloning. Oligonucleotide primers EA026 and EA027 were used to amplify the putative lactate-inducible promoter from *C. necator* H16 genomic DNA, and cloned into pBRC1 by AatII and NdeI restriction sites.

pEA007 was constructed by restriction enzyme-based cloning. Oligonucleotide primers EA026 and EA028 were used to amplify the putative lactate-inducible system from *C. necator* H16 genomic DNA, and cloned into pBRC1 by AatII and NdeI restriction sites.

pEA010 was constructed by restriction enzyme-based cloning. Oligonucleotide primers EA032 and EA033 were used to amplify the putative lactate-inducible promoter from *P. putida* KT2440 genomic DNA, and cloned into pBRC1 by AatII and NdeI restriction sites.

pEA011 was constructed by restriction enzyme-based cloning. Oligonucleotide primers EA032 and EA034 were used to amplify the putative lactate-inducible system from *P. putida* KT2440 genomic DNA, and cloned into pBRC1 by AatII and NdeI restriction sites.

pEA012 was constructed by restriction enzyme-based cloning. Oligonucleotide primers EA035 and EA037 were used to amplify the putative glycolic acid-inducible system from *P. putida* KT2440 genomic DNA, and cloned into pBRC1 by AatII and NdeI restriction sites.

pEA013 was constructed by restriction enzyme-based cloning. Oligonucleotide primers EA035 and EA038 were used to amplify the putative glycolic acid-inducible promoter from *P. putida* KT2440 genomic DNA, and cloned into pBRC1 by AatII and NdeI restriction sites.

pEA014 was constructed by using the NEBuilder Hifi DNA assembly method. Oligonucleotide primers EA013 and EA016 were used to amplify the putative lactate inducible promoter and *rfp* from pIE005. Oligonucleotide primers EG017 and N78_lact9_AscI_f were used to amplify the *iclR* from *C. necator* H16 genomic DNA with the core sequence of constitutive promoter P_{13}. The PCR product was combined with AscI/BamHI digested pBRC1.

pEA015 was constructed by using the NEBuilder Hifi DNA assembly method. Oligonucleotide primers EA045, EA041 and EA039, EA038 were used to amplify putative L-lactate inducible promoter and *lldR* from *E. coli* MG1655 genomic DNA with the core sequence of constitutive promoter P_{13}. The PCR product was combined with AatII/NdeI digested pBRC1.

pEA016 was constructed by restriction enzyme-based cloning. Oligonucleotide primers EA040 and EA041 were used to amplify putative L-lactate inducible promoter from *E. coli* MG1655 genomic DNA, and cloned into pBRC1 by AatII and NdeI restriction sites.

pEA015_60 was constructed by using the NEBuilder Hifi DNA assembly method. Oligonucleotide primers EA060C and EA38C were used to amplify *lldR* from pEA015 with a modified RBS sequence. The PCR product was combined with AatII/SpeI digested pBRC1.

pEA015_61 was constructed by using the NEBuilder Hifi DNA assembly method. Oligonucleotide primers EA061C and EA38C were used to amplify *lldR* from pEA015 with a modified RBS sequence. The PCR product was combined with AatII/SpeI digested pBRC1.

pEA015_62 was constructed by using the NEBuilder Hifi DNA assembly method. Oligonucleotide primers EA062B and EA38C were used to amplify *lldR* from pEA015 with a modified RBS sequence. The PCR product was combined with AatII/SpeI digested pBRC1.

pEA015_63 was constructed by using the NEBuilder Hifi DNA assembly method. Oligonucleotide primers EA063B and EA38C were used to amplify *lldR* from pEA015 with a modified RBS sequence. The PCR product was combined with AatII/SpeI digested pBRC1.

pEA017 was constructed by restriction enzyme-based cloning. Oligonucleotide primers EA042 and EA043 were used to amplify putative lactate inducible promoter from *Pseudomonas aeruginosa* PAO1 genomic DNA, and cloned into pBRC1 by AatII and NdeI restriction sites.

pEA018 was constructed by restriction enzyme-based cloning. Oligonucleotide primers EA042 and EA044 were used to amplify putative lactate inducible system from *E. coli* MG1655 genomic DNA, and cloned into pBRC1 by AatII and NdeI restriction sites.

pEA019 was constructed by using the NEBuilder Hifi DNA assembly method. Oligonucleotide primers EA046 and EA047 were used to amplify putative lactate inducible system from *C. necator* H16 genomic DNA, and cloned into pBRC1 by AatII and NdeI restriction sites.

pEA020 was constructed by using the NEBuilder Hifi DNA assembly method. Oligonucleotide primers EA047 and EA049 were used to amplify promoter *FadR* family transcriptional regulator from *C. necator* H16 genomic DNA. Oligonucleotide primers EA046 and EA048 were used to amplify putative lactate inducible promoter from *C. necator* H16 genomic DNA. The PCR product was combined with AatII/NdeI digested pBRC1.

pEA021 was constructed by using the NEBuilder Hifi DNA assembly method. Oligonucleotide primers EA050 and EA051 were used to amplify glycolic acid-inducible system from *C. necator* H16 genomic DNA. The PCR product was combined with AatII/NdeI digested pBRC1.

pEA022 was constructed by using the NEBuilder Hifi DNA assembly method. Oligonucleotide primers EA050 and EA052 were used to amplify putative glycolic acid-inducible promoter from *C. necator* H16 genomic DNA. The PCR product was combined with AatII/NdeI digested pBRC1.

pEA023 was constructed by using the NEBuilder Hifi DNA assembly method. Oligonucleotide primers EA053 and EA051 were used to amplify the putative glycolic acid-inducible promoter

from *C. necator* H16 genomic DNA. The PCR product was combined with AatII/NdeI digested pBRC1.

pEA024 was constructed by using the NEBuilder Hifi DNA assembly method. Oligonucleotide primers EA054 and EA056 were used to amplify the putative lactate inducible promoter from *P. fluorescens* NCTC 10038 genomic DNA. The PCR product was combined with AatII/NdeI digested pBRC1.

pEA025 was constructed by using the NEBuilder Hifi DNA assembly method. Oligonucleotide primers EA054 and EA055 were used to amplify putative lactate-inducible system from *P. fluorescens* NCTC 10038 genomic DNA. The PCR product was combined with AatII/NdeI digested pBRC1.

pEA026 was constructed by using the NEBuilder Hifi DNA assembly method. Oligonucleotide primers EA057 and EA058 were used to amplify the putative lactate inducible promoter from *P. lactis* DSM 29167 genomic DNA. The PCR product was combined with AatII/NdeI digested pBRC1.

pEA027 was constructed by using the NEBuilder Hifi DNA assembly method. Oligonucleotide primers EA057 and EA059 were used to amplify putative lactate-inducible system from *P. lactis* DSM 29167 genomic DNA. The PCR product was combined with AatII/NdeI digested pBRC1.

pEA028 was constructed by using the NEBuilder Hifi DNA assembly method. Oligonucleotide primers EA079 and EA078 were used to amplify the *iclR* from pIE005. Oligonucleotide primers EA077 and EA076 were used to amplify arabinose-inducible system from pBRC1. Oligonucleotide primers EA013 and EA016 were used to amplify the putative lactate inducible promoter and *rfp* from pIE005. The PCR product was combined with AscI/BamHI digested pBRC1.

pEA030 was constructed by using the NEBuilder Hifi DNA assembly method. Oligonucleotide primers EA086 and EA085 were used to amplify LysR family transcriptional regulator from *C. necator* H16 genomic DNA. Oligonucleotide primers EA087 and EA052 were used to amplify putative glycolic acid-inducible promoter from *C. necator* H16 genomic DNA. Oligonucleotide primers EA051 and EA053 were used to amplify putative glycolic acid-inducible promoter from *C. necator* H16 genomic DNA. The PCR product was combined with AatII/NdeI digested pBRC1.

pEA032 was constructed by using the NEBuilder Hifi DNA assembly method. Oligonucleotide primers EA089 and EA090 were used to amplify the putative lactate inducible system from *E. coli* MG1655 genomic DNA, and cloned into pBRC1 by AatII and NdeI restriction sites.

pEA033 was constructed by using the NEBuilder Hifi DNA assembly method. Oligonucleotide primers EA038 and EA081 were used to amplify the *lldR* from pEA015. Oligonucleotide primers EA088 and EA083 were used to amplify arabinose-inducible system from pBRC1. Oligonucleotide primers EA013 and EA084 were used to amplify the putative L-lactate inducible promoter and *rfp* from pIE005. The PCR product was combined with AscI/BamHI digested pBRC1.

The antibiotic resistance gene for plasmids pEA010, pEA011, pEA017, pEA018, pEA024, pEA025, pEA026, pEA027, pEA012, and pEA013 was changed to tetracycline. Oligonucleotide primers IK003 and IK004 were used to amplify the tetracycline resistance marker from pME6000, and cloned into pEA010, pEA011, pEA017, pEA018, pEA024, pEA025, pEA026, pEA027, pEA012, and pEA013 by PmeI and AscI restriction sites.

Supplementary Tables

Supplementary Table S1. Homology of proteins involved in the lactate catabolism.

Group[a]	Protein used for homology search	Protein sequence coverage and identity (%)[b]						
		*Ec*LldR/P_{lldP}	*Pp*PdhR/P_{lldP}	*Pa*PdhR/P_{lldP}	*Pf*PdhR/P_{LctP}	*Pl*PdhR/P_{LctP}	*Cn*GntR/$P_{H16\_RS19190}$	*Ec*GlcC/P_{b2979}
I	*Ec*LldP (b3603)	100 (100)	99 (66.37)	99 (65.18)	99 (65.48)	99 (64.95)	100 (65.13)	99 (63.08)
	*Ec*LldD (b3605)	100 (100)	98 (84.74)	95 (86.58)	ND	ND	ND	ND
	*Ec*LldR (b3604)	100 (100)	97 (41.09)	90 (41.91)	94 (40.32)	97 (40.16)	81 (34.86)	86 (31.20)
	*Pp*LldE (PP_4737)	ND	100 (100)	99 (81.69)	99 (84.49)	99 (84.92)	ND	ND
		*Cn*GntR/$P_{H16\_RS19190}$		*Ec*RclR/P_{b0306}			*Cn*IclR/$P_{H16\_RS06900}$	
II	*Cn*LldF (H16_RS19180)	100 (100)		97 (39.79)			97 (38.96)	
	*Cn*LldG (H16_RS19185)	100 (100)		40 (31.07)			ND	
	*Cn*LldE (H16_RS19190)	100 (100)		91 (34.85)			90 (40.51)	
	*Cn*GntR (H16_RS19195)	100 (100)		NS with *Ec*RclR			NS with *Cn*IclR; 67 (36.77) with *Cn*FadR	
		*Ec*GlcC/P_{b2979}		*Pp*GlcC/P_{PP_3745}			*Cn*LysR/$P_{H16\_RS15430}$	
III	*Ec*GlcD (b2979)	100 (100)		100 (80.76)			96 (61.20)	
	*Ec*GlcE (b2978)	100 (100)		100 (64.29)			96 (47.37)	
	*Ec*GlcF (b2978)	100 (100)		100 (72.73)			99 (55.05)	
	*Ec*GlcC (b2980)	100 (100)		95 (62.14)			NS with *Cn*LysR	

[a] groups were designated as in Fig. 1; [b] the protein sequence identity is indicated in brackets and the inducible systems, controlling expression of identified protein homologues, are shown above protein sequence coverage and identity percentage values; ND – protein homologue was not determined ; NS – no significant homology was found

Supplementary Table S2. Chemical compounds used in this study

Chemical	Producer	CAS Number
Sodium L-lactate, 98+%	Alfa Aesar, Thermo Fisher Scientific	867-56-1
Sodium D-lactate, >=99.0%	Sigma-Aldrich	920-49-0
3-hydroxypropanoic acid solution, 30%	Sigma-Aldrich	503-66-2
Sodium glyoxylate monohydrate	Sigma-Aldrich	918149-31-2
Sodium pyruvate	Sigma-Aldrich	113-24-6
Sodium glycolate	Alfa Aesar, Thermo Fisher Scientific	2836-32-0

Supplementary Table S3. Bacterial strains used in this study

Strain	Characteristic	Reference or source
Escherichia coli TOP10	F- *mcrA* Δ(*mrr-hsd*RMS-*mcr*BC)<br>Φ80*lac*ZΔM15<br>Δ *lac*X74 *rec*A1 *ara*D139<br>Δ(*araleu*)7697 *gal*U *gal*K *rps*L<br>(StrR) *end*A1 *nup*G	Invitrogen
Escherichia coli DH5α	F^- Φ80*lac*ZΔM15 Δ(*lac*ZYA-*arg*F)<br>U169 *rec*A1 *end*A1 *hsd*R17(r_k^-,<br>m_k^+) *pho*A *sup*E44 *thi*-1 *gyr*A96 *rel*A1 λ^-	Invitrogen
Escherichia coli MG1655	Wild type strain	DSM 18039
Cupriavidus necator H16	Wild type strain	DSM 428
Pseudomonas putida KT2440	Wild type strain	DSM 6125
Pseudomonas fluorescents NCTC10038	Wild type strain	DSM 50090
Pseudomonas lactic DSM29167	Wild type strain	DSM 29167

Supplementary Table S4. Oligonucleotide primers used in this study. Restriction sites used for cloning are underlined.

Primer name	Primer sequence (5’ → 3’)
EA021	atatatcatatgtaggcttcgctttgttgtgttgtgtg
EA022	atatgacgtctcccggacctcgtgcaca
EA023	atatgacgtccctaactcaggttcatctccagc
EA024	atatgacgtcataaaaccgccagaaatcaggg

EA025	atatatcatatgtaatactccattacttcatgcccat
EA026	atatatcatatgtcgctccggacgtgctg
EA027	atatgacgtcgaccgactccacgcgtc
EA028	atatgacgtctggtaggaccaatgcctgc
EA031	atatgacgtccgaaatctgcacaccttggg
EA032	ttgccaggtttgcatatggg
EA033	atatgacgtccacatgctctccacaaaccc
EA034	atatgacgtccggtactgcccataaaagcc
EA035	atatatcatatgctcactcgcaacggtttttg
EA037	atatggcgcgccataaaacgaaaggctcagtcgaaagactgggcctttcgttttatgacgtcgacgtccctgtggctgaccattgagt
EA038	gcctttcgttttatgacgtctcatgcgtttttctccctcgaatg
EA039	ttcccttttaatcatccggctcgtataatgtgtggagacttgaattcactagtttaactttaagaaggagatatatctaatgattgttttacccagacg
EA040	aggaatcatccacgttaaggacgtcctttaccagacatctccccccac
EA041	acgtcttcgctactcgccatatgaggtctcctggagtccacg
EA042	atatatcatatgggttggctccctaattgt
EA043	atatgacgtctcccgacacccttacccg
EA044	atatgacgtctagtcttcctgcacgctgc
EA045	gccggatgattaaaagggaactttaccagacatctccccccac
EA046	acgtcttcgctactcgccatatgcgacttctccagacccgaaac
EA047	gcctttcgttttatgacgtcaatggtggtgtcgtctcagg
EA048	gtctgcgtcggacatagatg
EA049	catctatgtccgacgcagacgcctgaaggccgaatcgc
EA050	gcctttcgttttatgacgtcgccggtctcctcgtaagc
EA051	acgtcttcgctactcgccatatgcgactcctgtggggcct
EA052	atatatcatatgggtctccggcgggtc
EA053	atatgacgtccgacatggtctgcttcctg
EA054	cgtcttcgctactcgccatatggggtggcccctaattg
EA055	gggcctttcgttttatgacgtctccttgtgacactgtagccttg
EA056	gggcctttcgttttatgacgtcttactgacgcacctgatcaaacc
EA057	cgtcttcgctactcgccatatggggtggcccctga
EA058	gggcctttcgttttatgacgtcttactgacgcacctgatcaaacc
EA059	gggcctttcgttttatgacgtcaatccttgtgacggtgtagc
EA060C	ggagacttgaattcactagtacaagcagttataaggaggttattttatgattgttttacccagacgc
EA061C	ggagacttgaattcactagtataagaagacttaaggacaaaattgtatgattgttttacccagacgc

EA062B	ggagacttgaattcactagtctttcacagctcatcaagaggaatccatgattgttttacccagacgc
EA063B	ggagacttgaattcactagttaaatacctattggacggagaagctcatgattgttttacccagacgc
EA076	tgtctgcgtcggacatatgtatatctccttcttaaaagatcttttgaattcc
EA077	catttctctttttccatctttaagcagaaggccatcctgacggatggcctttttgcgtttctacttatgacaacttgacggcta
EA078	atgtccgacgcagacaagt
EA079	ggcctttcgttttatgacgtc
EA081	atgattgttttacccagacgc
EA083	tctgggtaaaacaatcatatgtatatctccttcttaaaagatcttttgaattcc
EA084	agatatatctccttcttaaagttaaactagtg
EA085	gcctttcgttttatgacgtctaccagtccaggctcag
EA086	gacccgccggagacccatggcctccatgttcaacc
EA087	caggaagcagaccatgtcggtagaaacgcaaaaaggccatccgtcaggatggccttctgcttaagccggtctcctcgtaagc
EA088	cactagtttaactttaagaaggagatatatctgtagaaacgcaaaaaggccatccgtcaggatggccttctgcttaattatgacaacttgacggcta
EA089	ataacgtcttcgctactcgccatatgccactccttgtggtggc
EA090	gcctttcgttttatgacgtcatggatttttcttcctttgttgcc
IK003	tcgttttatggcgcgccaggccggccaattagaaggccgccagagagg
IK004	actagtactgtttaaaccgctcacaattccacacaa

Supplementary Figures

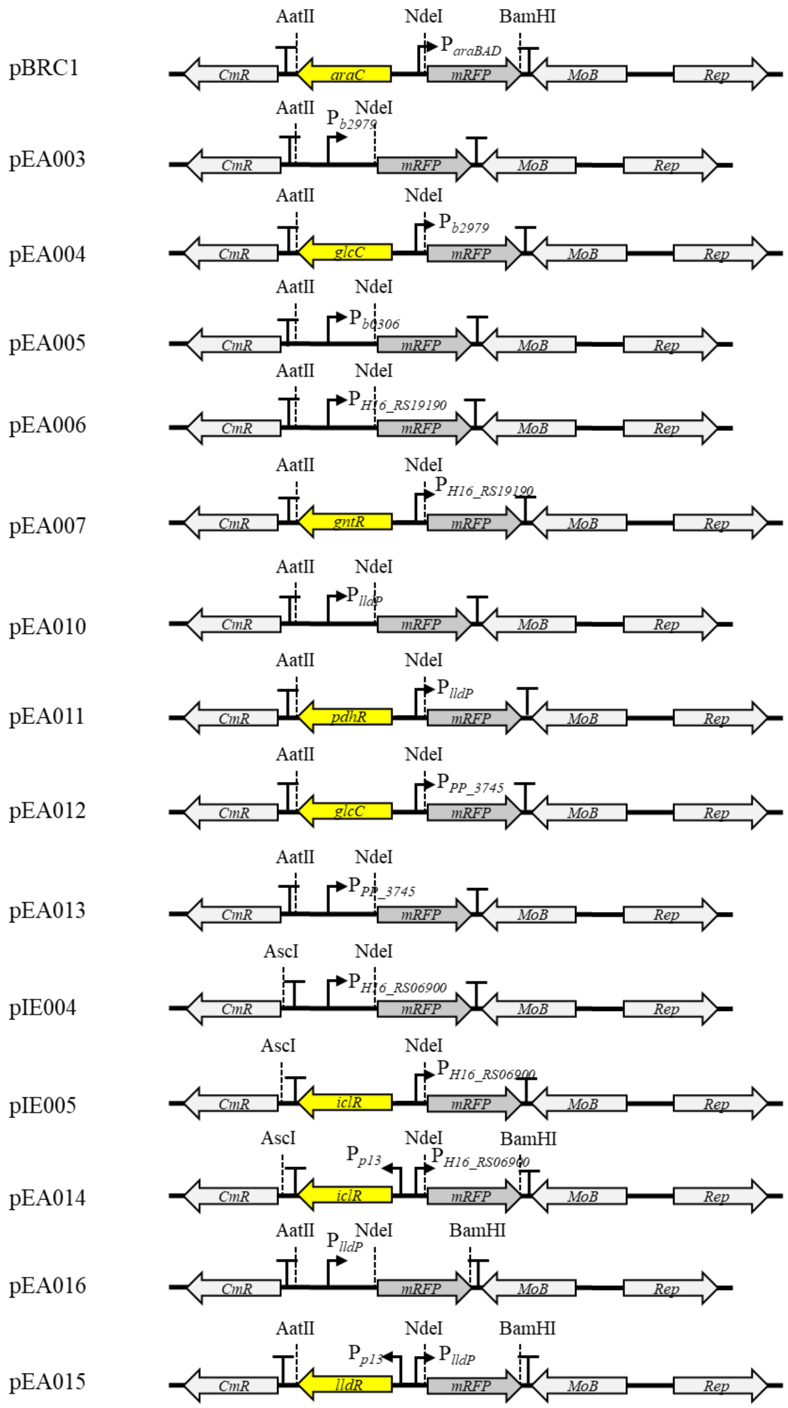

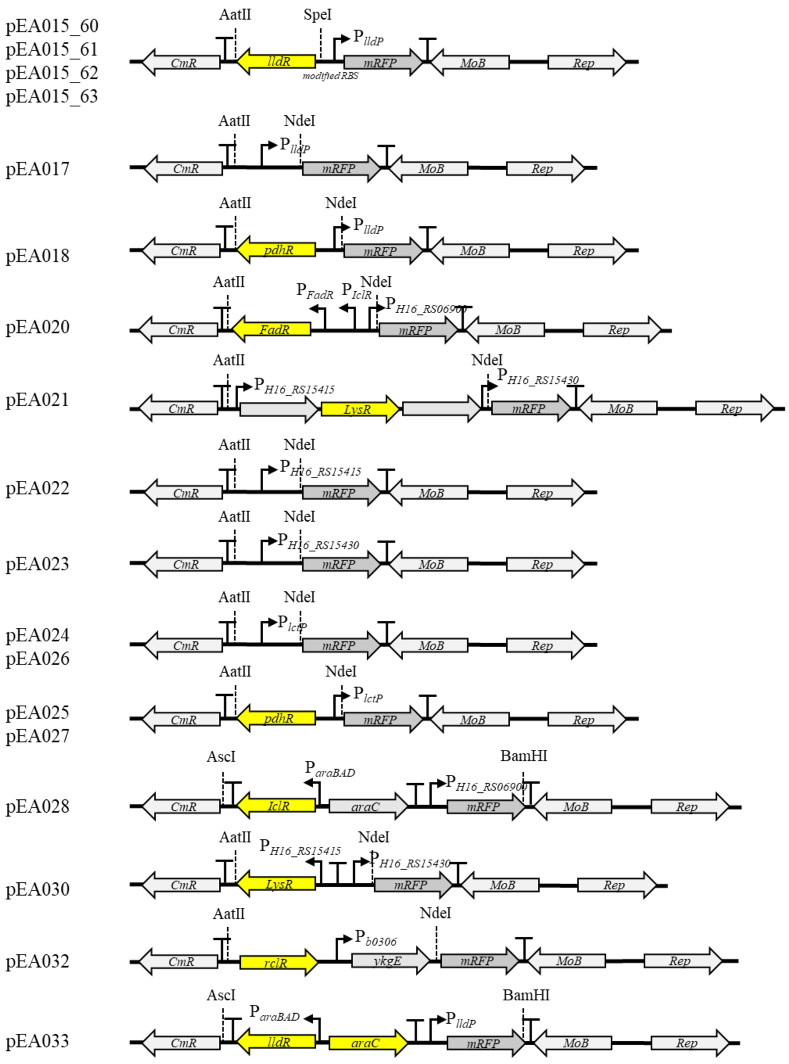

Supplementary Figure S1. Schematic illustration of plasmids constructed in this study. Here AatII, NdeI, AscI, SpeI and BamHI represent positions of restriction sites; *CmR*-chloramphenicol resistance gene; *mRFP*-red fluorescent protein gene; *MoB*- plasmid mobility protein gene; *Rep*-replication protein gene. Transcriptional regulator genes (in yellow), promoters (arrows), and terminators are indicated.

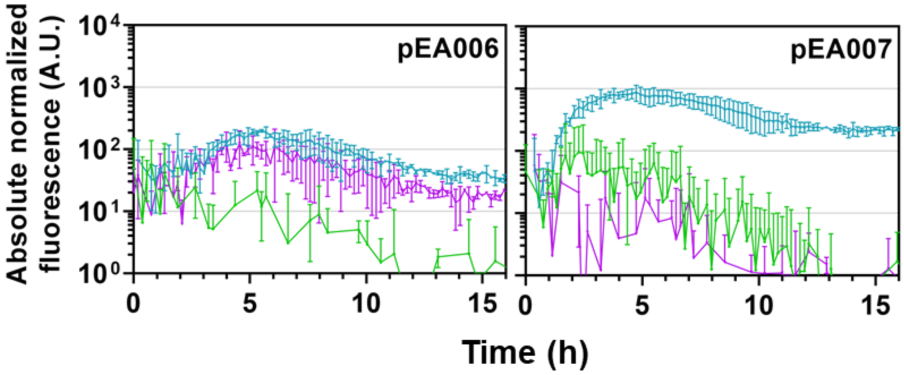

Supplementary Figure S2. Absolute normalized fluorescence of *C. necator* H16 harbouring constructs pEA006 and pEA007 with *Cn*P$_{H16_RS19190}$ and *Cn*GntR/P$_{H16_RS19190}$, respectively. Cells were grown in minimal medium containing 0.4% sodium gluconate for 16 hours. RFP-fluorescence output was determined in the absence of ligand (green) and in the presence of either the L-lactate (blue) or glycolic acid (purple), which were added at time 0 hour to a final concentration of 5 mM. Data are mean ± SD, *n* = 3.

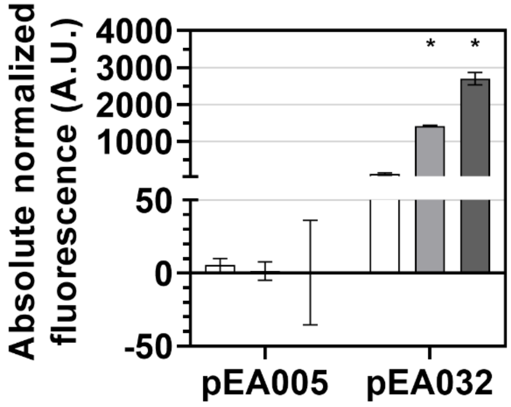

Supplementary Figure S3. Absolute normalized fluorescence of *E. coli* DH5*α* harbouring the constructs pEA005 and pEA032 with *Ec*P$_{b0306}$ and *Ec*RclR/P$_{b0306}$, respectively. Cells were grown with glucose (white), L-lactate (light grey), and D-lactate (dark grey) as carbon source. Single time-point fluorescence measurements were taken at 6 hour. Error bars represent standard deviations of three biological replicates. Asterisks indicate statistically significant induction values for *p < 0.01 (unpaired *t*-test).

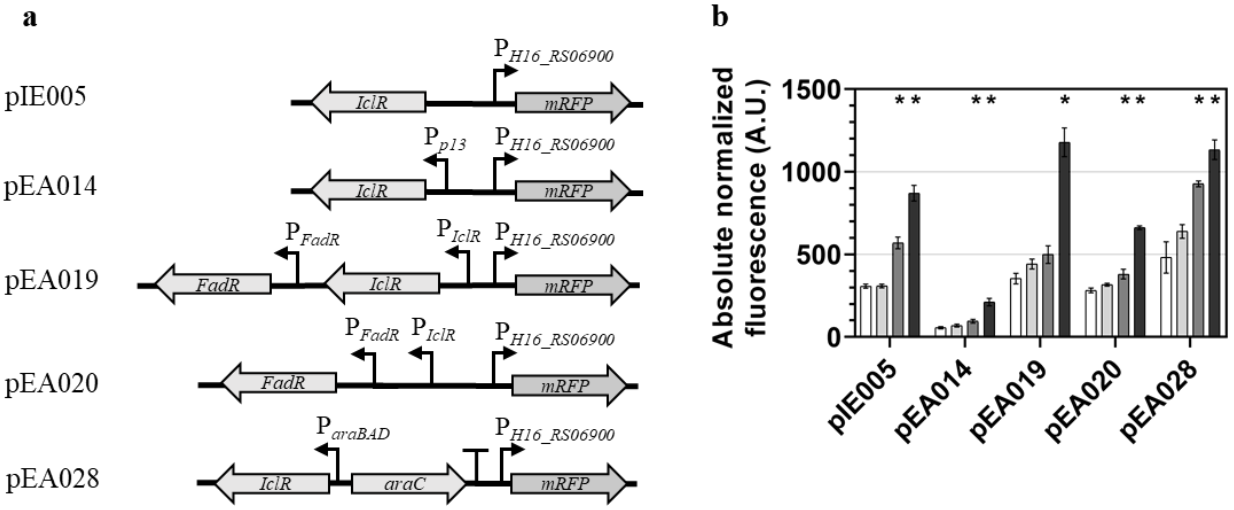

Supplementary Figure S4. Engineering the *Cn*IclR/P$_{H16_RS06900}$ system. (**a**) Schematic illustration of the different versions of the *Cn*IclR/P$_{H16_RS06900}$ system and their corresponding plasmid identifiers. (**b**) Absolute normalized fluorescence of *C. necator* H16 carrying different versions of the *Cn*IclR/P$_{H16_RS06900}$ system–reporter construct in the absence of inducer (white) and extracellular supplementation with L-lactate (light grey), D-lactate (dark grey), and glycolate (black) to a final concentration of 5mM. In the case of pEA028, the culture was also supplemented with 0.2% L-arabinose. Cells were grown in minimal medium containing 0.4% sodium gluconate. Error bars represent standard deviations of three biological replicates. Asterisks indicate statistically significant induction values for *p < 0.01 (unpaired *t*- test).

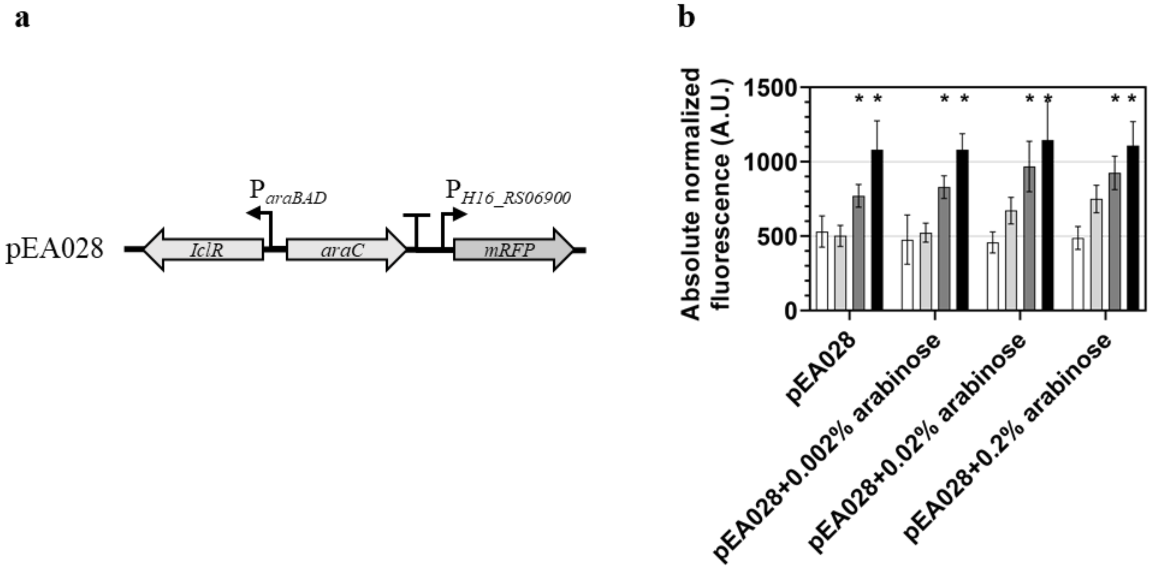

Supplementary Figure S5. (**a**) Schematic illustration of the *Cn*IclR/P$_{H16_RS06900}$ system with an arabinose-inducible system AraC/P$_{araBAD}$. (**b**) Absolute normalized fluorescence of *C. necator* H16

carrying of pEA028 vector with different arabinose concentration in the absence of inducer (white) and extracellular supplementation with L-lactate (light grey), D-lactate (dark grey), and glycolate (black) to a final concentration of 5mM. Cells were grown in minimal medium containing 0.4% sodium gluconate. Error bars represent standard deviations of three biological replicates. Asterisks indicate statistically significant induction values for *p < 0.01 (unpaired *t*-test).

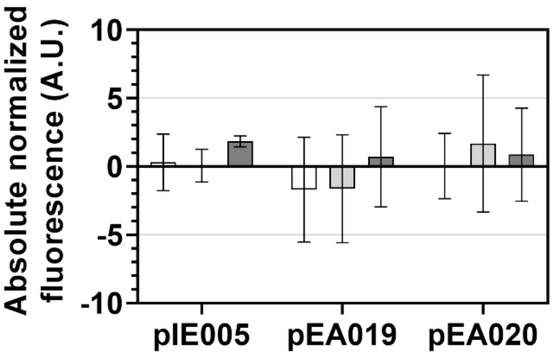

Supplementary Figure S6. Absolute normalized fluorescence of *E. coli* MG1655 harbouring constructs pIE005, pEA019, and pEA020 with *Cn*IclR/$P_{H16\_RS06900}$, *Cn*IclRFadR/$P_{H16\_RS06900}$, and *Cn*FadR/$P_{H16\_RS06900}$, respectively. RFP fluorescence output was determined in the absence of inducer (white) and 6 h after extracellular supplementation with D-lactate (light grey) and glycolate (dark grey) to a final concentration of 5 mM. Cells were grown in minimal medium containing 0.4% glucose. Error bars represent standard deviations of three biological replicates.

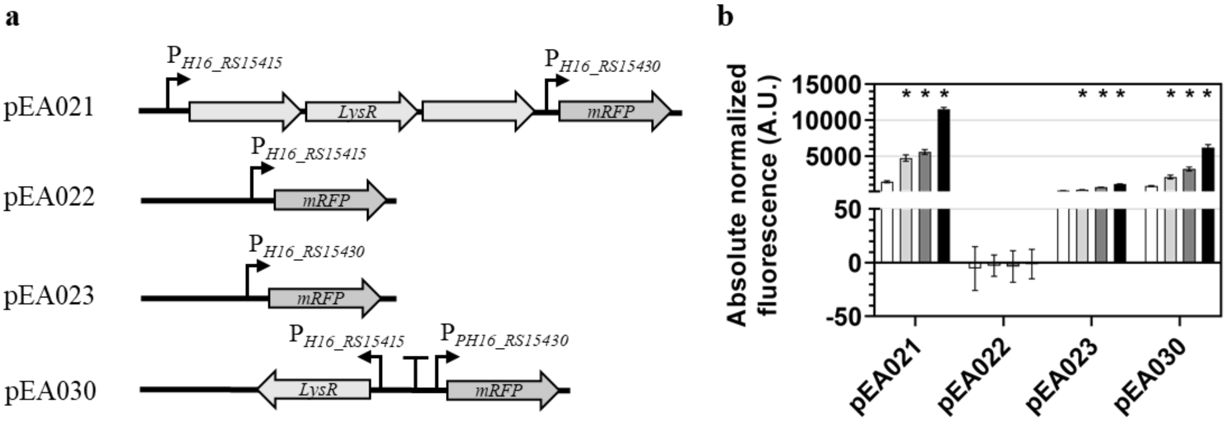

Supplementary Figure S7. Identification and characterization of the *Cn*LysR/$P_{H16\_RS15430}$ system. (**a**) Schematic illustration of the different versions of the *Cn*LysR/$P_{H16\_RS15430}$-inducible system and their corresponding plasmid identifiers. (**b**) Absolute normalized fluorescence of *C. necator* H16 carrying different versions of the inducible system/reporter construct without (white) and with extracellular supplementation of either L-lactate (light grey), D-lactate (dark grey), or glycolate

(black) to a final concentration of 5mM. Cells were grown in minimal medium containing 0.4% sodium gluconate. Error bars represent standard deviations of three biological replicates. Asterisks indicate statistically significant induction values for *p < 0.01 (unpaired *t*-test).

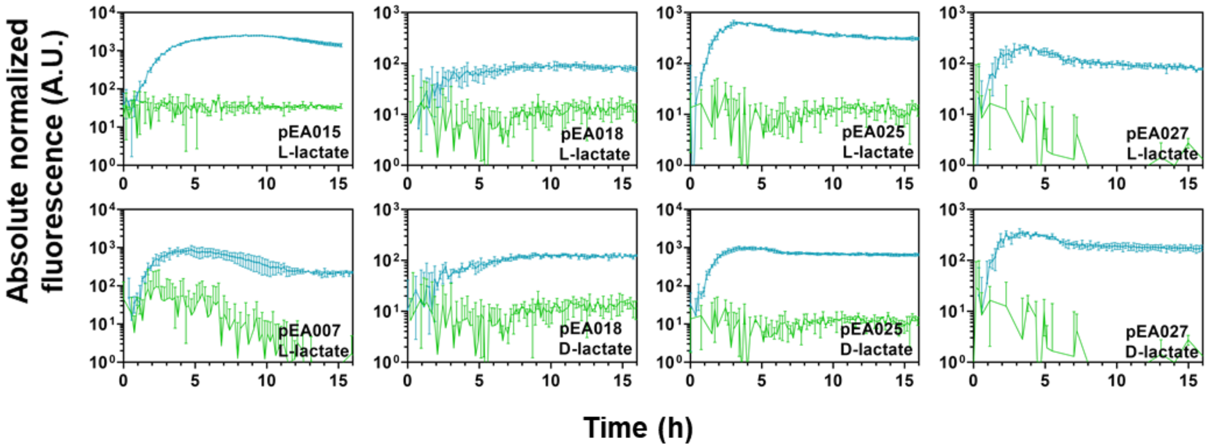

Supplementary Figure S8. Induction kinetics of the identified lactate-inducible systems. Absolute normalized fluorescence of *E. coli* DH5α harbouring pEA015 with *Ec*LldR/P_{lldP}, *C. necator* H16 harbouring pEA007 with *Cn*GntR/$P_{H16\_RS19190}$, and *P. putida* KT2400 harbouring pEA018, pEA025, and pEA027 with *Pa*PdhR/P_{lldP}, *Pf*PdhR/P_{lctP}, and *Pl*PdhR/P_{lctP}, respectively. Cells were grown in minimal medium containing 0.4% glucose (in the case of *C. necator* H16 supplemented with 0.4% gluconate) for 16 hours. RFP-fluorescence output was determined in the absence of inducer (green) and in the presence of either the L- or D-lactate (blue), which were added at time 0 hour to a final concentration of 5 mM. Data are mean ± SD, *n* = 3.

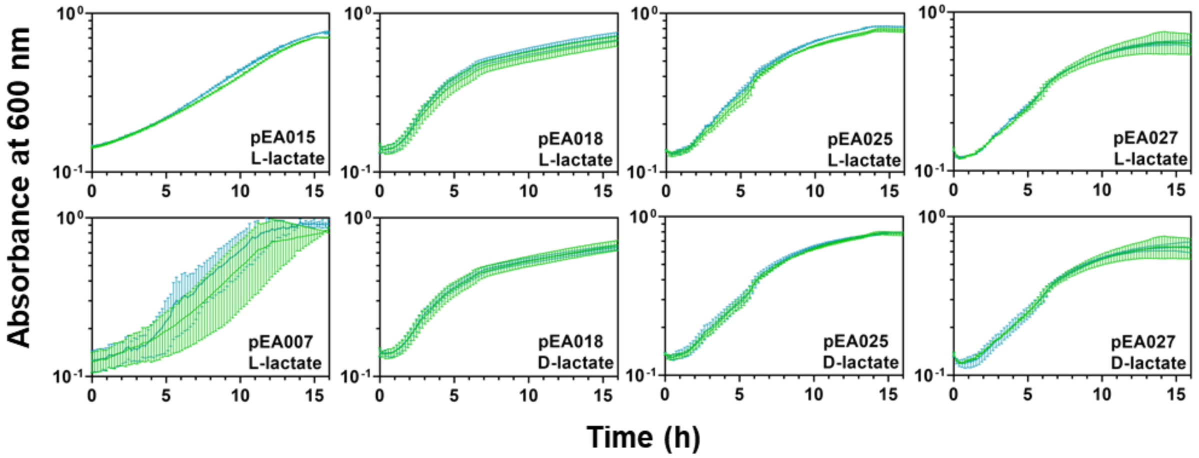

Supplementary Figure S9. Cell growth dynamics. Graphs represent the optical density over time in cell cultures of *E. coli* DH5α harbouring pEA015 with *Ec*LldR/P_{lldP}, *C. necator* H16 harbouring

pEA007 with *Cn*GntR/$P_{H16\_RS19190}$, and *P. putida* KT2400 harbouring constructs pEA018, pEA025, and pEA027 with *Pa*PdhR/P_{lldP}, *Pf* PdhR/P_{lctP}, and *Pl*PdhR/P_{lctP}, respectively. Cells were grown in minimal medium containing 0.4% glucose (in the case of *C. necator* H16 supplemented with 0.4% gluconate) for 16 hours. Absorbance was measured in the absence of inducer (green) and in the presence of either the L- or D-lactate (blue), which were added at time 0 hour to a final concentration of 5 mM. Data are mean ± SD, n = 3.

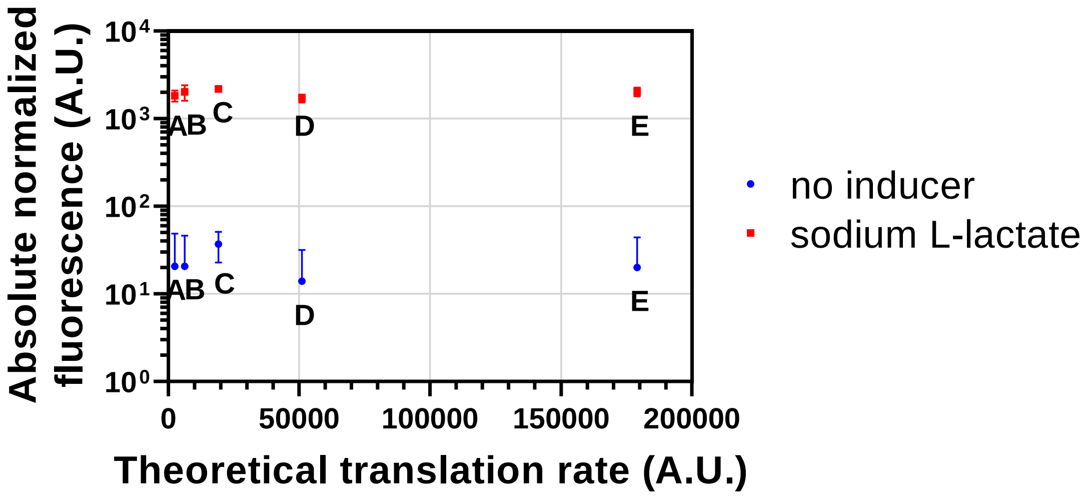

Supplementary Figure S10. Influence of *lldR* theoretical translation rate on the dynamic range of the *Ec*LldR/P_{lldP}-inducible system. Absolute normalized fluorescence values of *E. coli* DH5α carrying variants of the *Ec*LldR/P_{lldP}-inducible system with different RBS sequences upstream to the *lldR* gene are compared to the corresponding theoretical translation rates. Plasmid constructs containing *Ec*LldR/P_{lldP}-inducible system with different RBS sequence variants were as following: A-pEA015_63; B-pEA015_62; C-pEA015; D-pEA015_61; E-pEA015_60. Cells were grown in minimal medium containing 0.4% glucose. Data are mean ± SD, n =3.

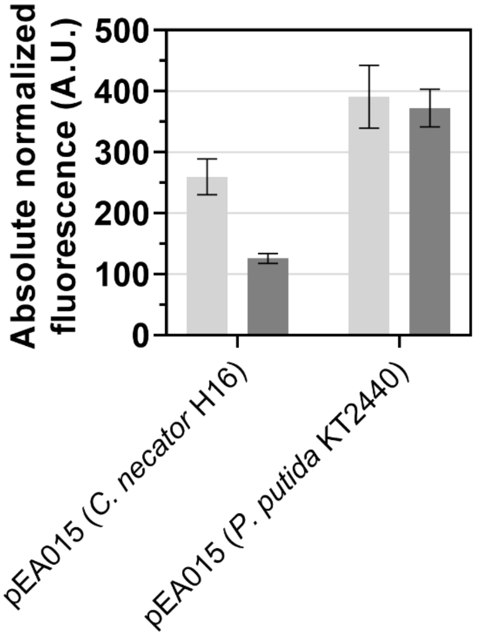

Supplementary Figure S11. Absolute normalized fluorescence of *C. necator* H16 and *P. putida* KT2440 carrying the *Ec*LldR/P_{lldP}-inducible gene expression system. RFP fluorescence output was determined in the absence of ligand (light grey) and 6 h after extracellular supplementation with L-lactate to a final concentration of 5mM (dark grey). Cells were grown in minimal medium containing 0.4% glucose (in the case of *C. necator* H16 supplemented with 0.4% gluconate). Data are mean ± SD, n =3.

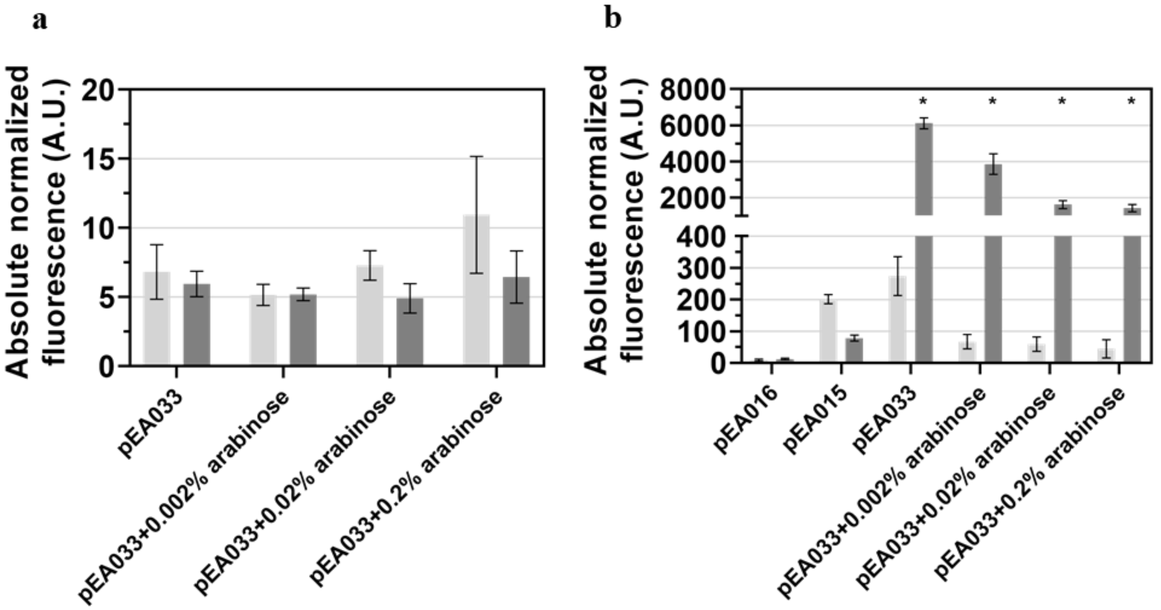

Supplementary Figure S12. Absolute normalized fluorescence of *C. necator* H16 harbouring different versions of the L-lactate-inducible system (*Ec*LldR/P_{lldP})**. (a)** Cells were grown in minimal medium containing 0.4% sodium gluconate in the absence of inducer (light grey) and extracellular supplementation with L-lactate to a final concentration of 5 mM (dark grey). **(b)** Cells were grown with gluconate (light grey) and L-lactate (dark grey) as carbon sources. In the case of pEA033, the culture was supplemented with different concentrations of L-arabinose as indicated. Single time-point fluorescence measurements were taken at 6 hour. Data are mean ± SD, n = 3, *p ≤ 0.01 (unpaired *t*-test).

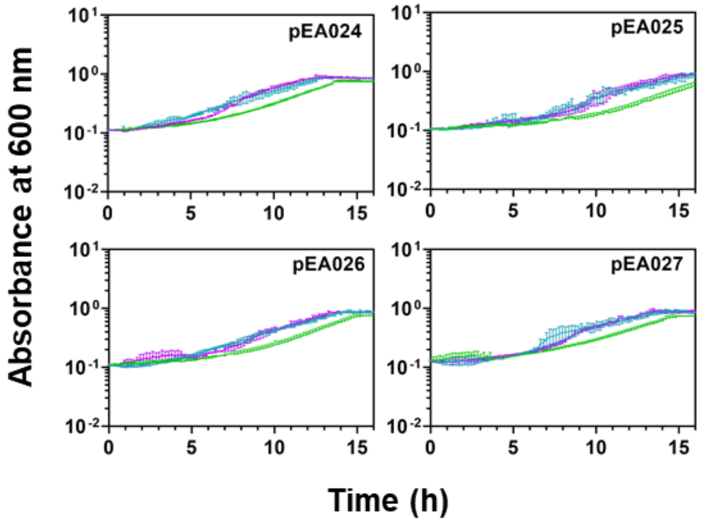

Supplementary Figure S13. Cell growth dynamics. Graphs represent the optical density over time in cell cultures of *C. necator* H16 harbouring pEA024, pEA025, pEA026, and pEA027 with *Pf*P_{LctP}, *Pf*PdhR/P_{lctP}, *Pl*P_{LctP}, and *Pl*PdhR/P_{lctP}, respectively. Cells were grown in minimal medium containing 0.4% gluconate for 16 hours. Absorbance was measured in the absence of inducer (green) and in the presence of the L-lactate (blue) and D-lactate (purple), which were added at time 0 hour to a final concentration of 5 mM. Data are mean ± SD, n = 3.

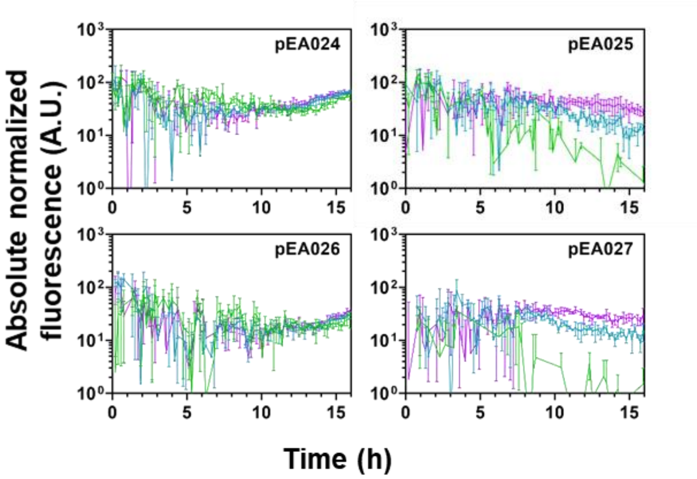

Supplementary Figure S14. Induction kinetics of the identified lactate-inducible systems. Absolute normalized fluorescence of *C. necator* H16 harbouring pEA024, pEA025, pEA026, and pEA027 with *Pf*P_{LctP}, *Pf*PdhR/P_{lctP}, *Pl*P_{LctP} and *Pl*PdhR/P_{lctP}, respectively. Cells were grown in minimal medium containing 0.4% gluconate for 16 hours. RFP-fluorescence output was determined in the absence of inducer (green) and in the presence of the L-lactate (blue) and D-lactate (purple), which were added at time 0 hour to a final concentration of 5 mM. Data are mean ± SD, $n = 3$.

References

[1] Hanko, E. K. R., Minton, N. P. and & Malys, N. Characterisation of a 3-hydroxypropionic acid-inducible system from *Pseudomonas putida* for orthogonal gene expression control in *Escherichia coli* and *Cupriavidus necator*. *Sci. Rep.* **7**, 1724 (2017).
